# Conformational analysis of macrocycles: comparing general and specialized methods

**DOI:** 10.1007/s10822-020-00277-2

**Published:** 2020-01-21

**Authors:** Gustav Olanders, Hiba Alogheli, Peter Brandt, Anders Karlén

**Affiliations:** 1 Department of Medicinal Chemistry, Uppsala University, BMC, Box 574, 751 23 Uppsala, Sweden; 2 Present Address: Medicinal Chemistry, Research and Early Development Cardiovascular, Renal and Metabolism, BioPharmaceuticals R&D, AstraZeneca, Gothenburg, Sweden

**Keywords:** Macrocycles, Conformational sampling, Drug design

## Abstract

**Abstract:**

Macrocycles represent an important class of medicinally relevant small molecules due to their interesting biological properties. Therefore, a firm understanding of their conformational preferences is important for drug design. Given the importance of macrocycle-protein modelling in drug discovery, we envisaged that a systematic study of both classical and recent specialized methods would provide guidance for other practitioners within the field. In this study we compare the performance of the general, well established conformational analysis methods Monte Carlo Multiple Minimum (MCMM) and Mixed Torsional/Low-Mode sampling (MTLMOD) with two more recent and specialized macrocycle sampling techniques: MacroModel macrocycle Baseline Search (MD/LLMOD) and Prime macrocycle conformational sampling (PRIME-MCS). Using macrocycles extracted from 44 macrocycle-protein X-ray crystallography complexes, we evaluated each method based on their ability to (i) generate unique conformers, (ii) generate unique macrocycle ring conformations, (iii) identify the global energy minimum, (iv) identify conformers similar to the X-ray ligand conformation after Protein Preparation Wizard treatment (X-ray_ppw_), and (v) to the X-ray_ppw_ ring conformation. Computational speed was also considered. In addition, conformational coverage, as defined by the number of conformations identified, was studied. In order to study the relative energies of the bioactive conformations, the energy differences between the global energy minima and the energy minimized X-ray_ppw_ structures and, the global energy minima and the MCMM-Exhaustive (1,000,000 search steps) generated conformers closest to the X-ray_ppw_ structure, were calculated and analysed. All searches were performed using relatively short run times (10,000 steps for MCMM, MTLMOD and MD/LLMOD). To assess the performance of the methods, they were compared to an exhaustive MCMM search using 1,000,000 search steps for each of the 44 macrocycles (requiring ca 200 times more CPU time). Prior to our analysis, we also investigated if the general search methods MCMM and MTLMOD could also be optimized for macrocycle conformational sampling. Taken together, our work concludes that the more general methods can be optimized for macrocycle modelling by slightly adjusting the settings around the ring closure bond. In most cases, MCMM and MTLMOD with either standard or enhanced settings performed well in comparison to the more specialized macrocycle sampling methods MD/LLMOD and PRIME-MCS. When using enhanced settings for MCMM and MTLMOD, the X-ray_ppw_ conformation was regenerated with the greatest accuracy. The, MD/LLMOD emerged as the most efficient method for generating the global energy minima.

**Graphic abstract:**

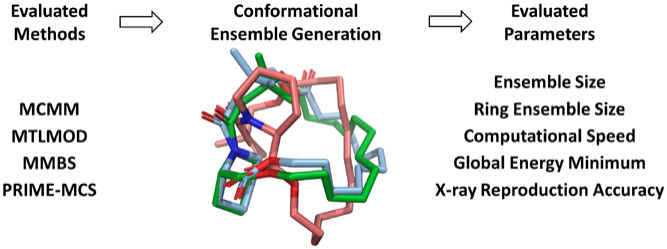

**Electronic supplementary material:**

The online version of this article (10.1007/s10822-020-00277-2) contains supplementary material, which is available to authorized users.

## Introduction

Computational modelling has transformed the strategic decision making process in drug discovery; both reducing costs and improving efficiency [1]. Prominent areas of contribution include pharmacophore-based, and shaped-based virtual screening [2-4], and docking [5] of drug candidates to their protein targets. Although these methods use different approaches, they all require conformational data as an input. Conformational sampling is also required for other computational techniques employed in medicinal chemistry, for example drug design [6], drug permeability prediction [7], NMR data interpretation [8-11], and fitting molecules to X-ray electron density maps [12]. Therefore, it is of great importance to have reliable and efficient methods for conformer generation.

Recently, macrocycles (herein defined as cyclic compounds with a ring size of 10 atoms or more) have gained increased importance in drug discovery because of their unique properties [6, 13-17]. Macrocycles may possess cell permeability better than expected by the “rule-of-five” [6, 17, 18], improved metabolic stability [11], enhanced binding properties to featureless binding sites [19], as well as the ability to disrupt protein–protein interactions [19-22]. However, macrocycles often present a significant synthetic challenge [13, 23-25]. It is therefore of great importance to develop and improve computational methods, such as conformational analysis, to focus the design of new macrocyclic ligands [26, 27].

In 1990, Saunders et al*.* performed a conformational analysis study on cycloheptadecane, aiming to identify the best method for searching large ring structures [28]. After evaluating systematic and random search methods, as well as molecular dynamics and a distance geometry method, they concluded that cycloheptadecane was lying at the boundary of what could be addressed with the technology of the time. In recent times, many new algorithms for exploring molecular potential energy surfaces have been developed e.g. LMOD [8], LLMOD [29], MTLMOD [30], LowModeMD [31], MD/LLMOD [32], PRIME-MCS [33], ForceGen [34], BRIKARD [35], PLOP [36], a DFT-D3/COSMO-RS based method [37], and, most recently, Conformator [38]. However, conformational sampling of macrocycles is still considered a challenging task [36, 39]. To provide guidance for other practitioners within the field we compare the conformational search capabilities of four different methods with respect to sampling the conformational space of macrocycles.

**Fig. 1:**
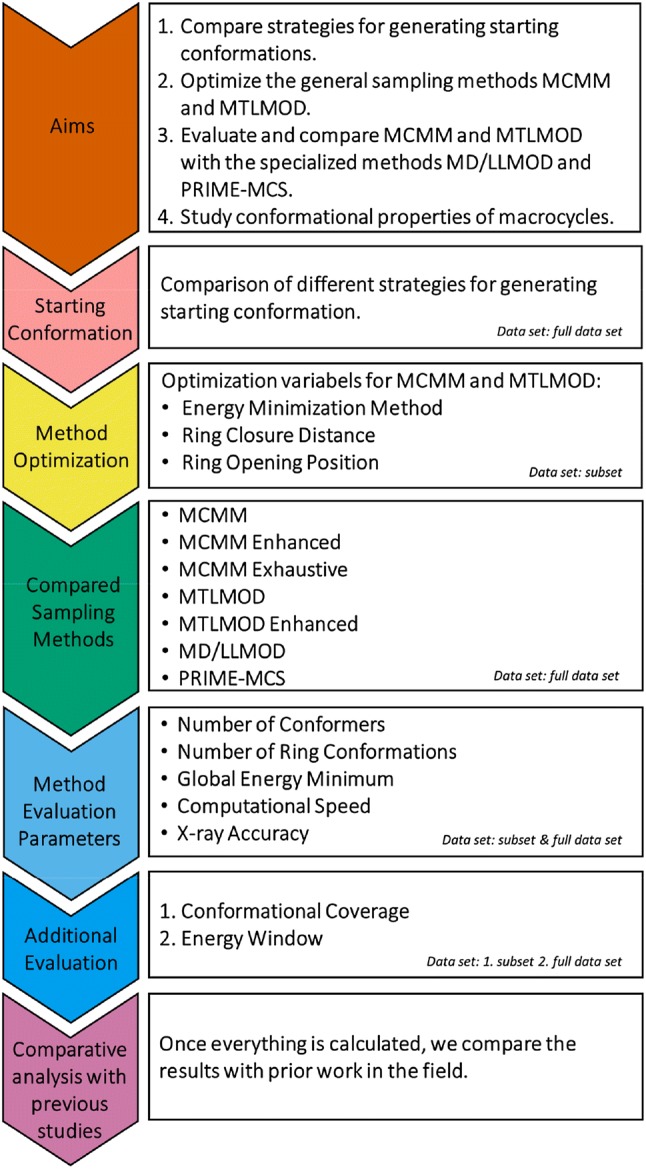
A graphical summary of the study design

In the current study, we use a data set of 44 protein-macrocycle complexes (38 unique ring systems) [40], where the majority of the structures originated from the commonly used data set of Watts et al. [32] In terms of sampling methods, we decided to include the general Monte Carlo Multiple Minimum (MCMM) method since it has not yet been extensively applied towards macrocycle sampling. The MCMM algorithm was published by Chang et al*.* in 1989 [41] and is implemented in the Schrödinger software MacroModel. In 1989, yet another conformational search algorithm called random incremental pulse search (RIPS) was published by Ferguson and Raber [42]. Today, a similar approach to RIPS, called stochastic search, is implemented in the Chemical Computing Group's Molecular Operating Environment (MOE) software [43]. The MCMM and MOE stochastic search methods are not built upon the same search algorithm and, therefore, we expect differences in their performance. Whilst the MOE stochastic search algorithm has often been used in conformational analysis comparison studies, MCMM has not been utilized in this capacity. Thus, we wanted to investigate the performance of MCMM applied on macrocycles. Another more general method, the Mixed Torsional/Low-Mode (MTLMOD) sampling conformational search method, was also included in this study. This method has been used in several recent publications to sample the conformational space of both macrocycles and non-macrocyclic structures [44]. Finally, we wanted to compare these more general conformational search methods with two more recent specialized macrocycle sampling methods: MacroModel macrocycle Baseline Search (MD/LLMOD) [32] and Prime macrocycle conformational sampling (PRIME-MCS) [33]. MD/LLMOD combines a short molecular dynamics simulation with Large-Scale Low-Mode steps. In comparison, PRIME-MCS splits the macrocycle backbone into two pieces, sampling them independently using predefined angle libraries before reconnecting the pieces again [33]. Before performing the method comparison, we investigated if the general methods (MCMM and MTLMOD) could also be optimized for macrocycle conformational sampling. Based on the findings in the optimization step, we performed the method comparison study where all search methods (including both standard and enhanced MCMM and MTLMOD) were benchmarked against an exhaustive MCMM search using 1,000,000 search steps. After comparatively evaluating these methods, we addressed the conformational coverage, as well as the energy difference between the conformer closest to the X-ray_ppw_ conformation and the “global energy minimum”. The workflow of the study is summarized in Fig. 1.

## Methods

Unless otherwise stated, all calculations were performed within the Schrödinger Small-Molecule Drug Discovery Suite 2017–1 [45] using the OPLS3 [46] force field with the GB/SA continuum solvation model for water [47]. When setting up a calculation in the Maestro GUI, some methods use kJ mol^−1^ and others kcal mol^−1^. Therefore, we decided to present the settings in the unit used, along with the alternative unit in parenthesis. All graphs presented herein were made in Python [48] and R [49]. Figures were made in Microsoft PowerPoint [50]. PCA-models (including score and loading plots) were made in SIMCA [51]. Molecular modeling figures were made in PyMOL [52].

### Data set selection

The macrocycles in the 47 protein–ligand complexes previously published by Alogheli et al*.* [40] were used in the present study. The three duplicate structures 2IYF, 1FKJ and, 1YET (Erythromycin in 2IYF/3FRQ and Tacrolimus in 1FKJ/4NNR, and Geldanamycin in 1YET/2ESA) present in the Alogheli data set, were removed from the data set to give 44 macrocycles.

### X-ray structures preparation

All 44 X-ray structures were downloaded from the Protein Data Bank (PDB) [53, 54] and prepared using the Protein Preparation Wizard [55, 56] in Maestro [57] using default options as described below. In the previous docking study by Alogheli et al. [40] the OPLS-2005 force field was used for preparing the structures while in the present work we used the OPLS3 force field. The PDB structures were therefore reprocessed using OPLS3. Missing side-chains were added by Prime side-chain predictions [58-60]. In cases where residues had alternate positions, the first listed position, or the position with the highest average occupancies, was selected. The ligand tautomer and ionization state, as well as protein protonation states, were the same as used in the study by Alogheli et al*.* [40] (see the tautomer and ionization state of the structures in Table 1). Furthermore, the hydrogen bond networks of the protein–ligand complexes were optimized and water molecules forming less than three hydrogen bonds to non-waters were removed. Finally, the protein–ligand complexes were energy minimized using default settings where heavy atoms were displaced no more than 0.3 Å Root Mean Square Deviation (RMSD).

**Table 1:**
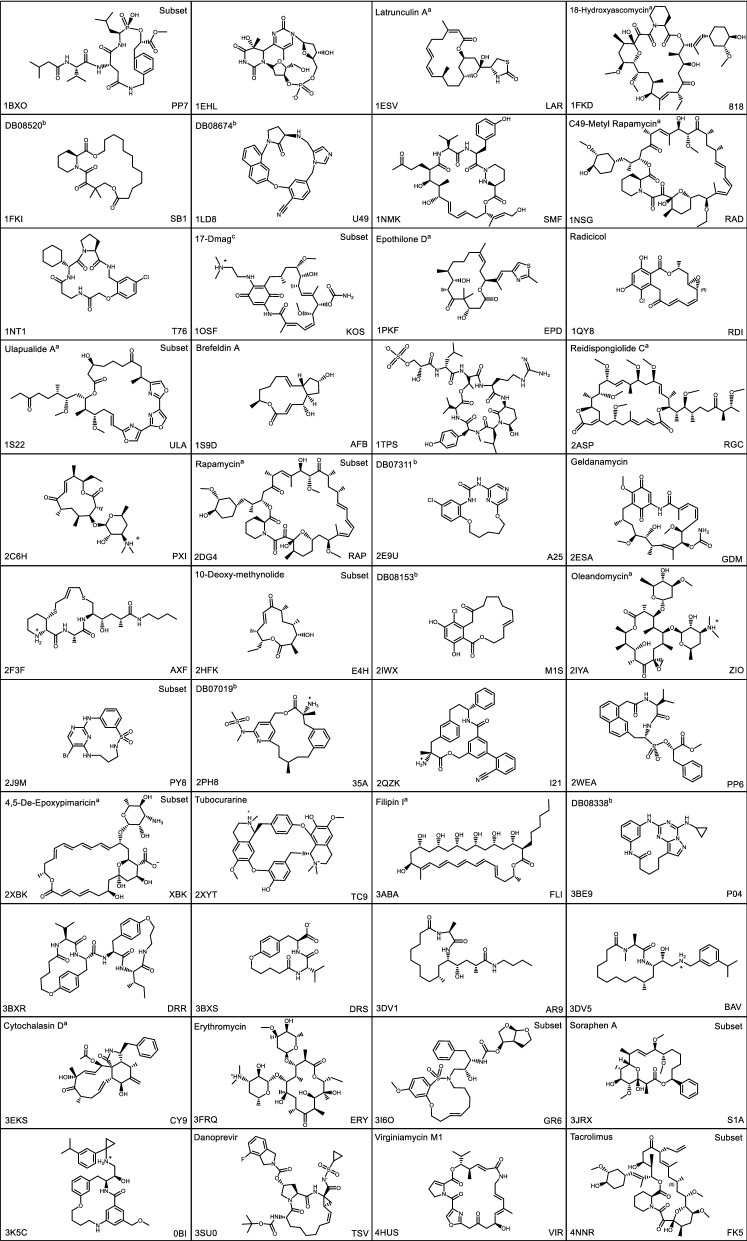
Structures of the Macrocycles in the Tautomer/Ionization States Used for Conformational Analysis

The PDB code of the complex structure are shown to the lower left, whereas the ligand name (when given) and the ligand code are shown to the upper left and lower right, respectively. Macrocycles that were included in the subset are marked with “subset” to the upper right.

### Selecting a non-biased starting conformation

To compare strategies for generating a non-biased starting conformation, two approaches were used. The most commonly employed method involves converting the X-ray structure to SMILES format, whilst retaining stereochemical information, and then converting it back to the 3D structure. Accordingly, all macrocycle X-ray structures in the current study were converted to SMILES codes and then back to their 3D structures using LigPrep, before these conformational geometries were compared to their corresponding X-ray structures. Alternatively, we also applied a more elaborate approach where we performed an MCMM conformational sampling of each macrocycle (starting from the SMILES generated structure) using 10,000 search steps and an energy window for keeping conformers of 62.8 kJ mol^−1^ (15.01 kcal mol^−1^). The conformer with the highest RMSD to the X-ray conformation was selected and further analyzed (hereafter called “starting conformer”). To compare the two strategies, we used a conformational clustering tool and calculated the torsional RMSD, including all dihedral angles except those involving terminal atoms, i.e. methyl hydrogens. The two strategies were also compared by calculating the number of torsional angles that deviated more than 120° or 60°, respectively, from the torsional angles found in the X-ray_ppw_ conformation (excluding terminal atom dihedral angles). We also included a comparison to the energy minimized X-ray_ppw_ structure. All calculations were performed by running a script in the command script editor followed by a separate python script.

The conformer most dissimilar to the X-ray_ppw_ structure after an MCMM search was selected as the “starting conformer” and was used for all conformational sampling studies performed in this study. A similar approach for generating the starting conformation was used by Coutsias et al*.* [35]

### Conformational sampling

All methods except MD/LLMOD and MCMM-Exhaustive were run in triplicates using different seeds.

#### Conformational sampling using MCMM and MTLMOD

The Monte Carlo Multiple Minimum (MCMM) and the Mixed torsional/Low-Mode sampling (MTLMOD) search methods implemented in MacroModel [61] were run with 10,000 steps in total for each compound. The option of using a fixed number of steps per rotatable bond, as well as the Multi-Ligand option, were deselected. Torsional sampling of amides, esters, as well as all C–N and C–O single bonds and C=N and N=N double bonds, were allowed in the search (“extended sampling”). For energy minimizations, up to 50,000 steps of Truncated Newton minimization (TNCG) [62] with a gradient convergence criterion of 0.05 kJ Å^−1^ mol^−1^ was used (the minimization terminates when the convergence criterion is met). A 0.5 Å distance threshold between any pair of heavy atoms (and O–H, S–H) was used for elimination of redundant conformers. The energy window for keeping conformers was set to 62.8 kJ mol^−1^ (15.01 kcal mol^−1^). For MTLMOD, the probability of a torsion rotation/molecule translation was set to the default value of 0.5. Also, the minimum and maximum distance for low-mode moves were kept at default values of 3.0 and 6.0 Å, respectively. Random seeding was achieved by modifying the .com files, see section “Random seeding for MCMM and MTLMOD” in supporting information.

#### Conformational sampling using one million search steps (MCMM-Exhaustive)

In this MCMM search the same settings as metioned above were used except that stereocenters adjacent to ring closures were avoided and a wider ring-opening criterion was used (0–100 Å). This search will be referred to as MCMM-Exhaustive and 1,000,000 search steps were used for each compound.

#### Conformational sampling using MD/LLMOD

For MacroModel Macrocycle Baseline Search (MD/LLMOD) the energy window for keeping conformers was set to 15.01 kcal mol^−1^ (62.8 kJ mol^−1^) and the torsion sampling option was set to extended mode, enabling ester and amide sampling. The remaining settings were left at their default values: elimination of redundant conformers using an RMSD of 0.75 Å, 5000 molecular dynamics simulation cycles and 5000 LLMOD (Large-scale Low-mode) search steps. Eigenvectors were determined for each new global minimum.

#### Conformational sampling using PRIME-MCS

PRIME-MCS was run from the command line. In short, PRIME-MCS was run in vacuum. PRIME-MCS was run using the sampling intensity “thorough” generating up to 1000 conformations. For more details about the used PRIME-MCS syntax, see “PRIME-MCS sampling-syntax” section in supporting information.

### Exploring energy minimization method and ring closure settings on a diverse subset of 10 macrocycles

#### Selection of a diverse subset

Ten diverse macrocycles were chosen from the 44 macrocycles to represent the full data set of 44 macrocycles using a principal component analysis (see section “Selection of a Diverse Subset.” in supporting information). The macrocycles in the subset are marked with “subset” in Table 1.

#### Conformational sampling of the macrocycles in the subset using MCMM and MTLMOD

The same settings as described above using 10,000 search steps was used except that this study was performed using only one seed.

#### Minimization method

The PRCG and TNCG minimization methods were compared using the MCMM and MTLMOD methods. For energy minimizations, up to 50,000 steps of PRCG or TNCG minimization with a gradient convergence criterion of 0.05 kJ Å^−1^ mol^−1^ was used (the minimization terminates when the convergence criterion is met).

#### Ring closure criterion

The default ring closure criterion closes the opened ring systems if the distance between the ring-opened atoms are between 0.5 and 2.5 Å. It is recommended to use a wider ring closure criterion of ca. 0.1–5.0 Å for larger ring systems, therefore, this distance was evaluated [63]. A very wide ring closure criteria, between 0–100 Å, was also evaluated.

### Evaluation of the conformational search methods

The performance of the conformational search methods were evaluated with respect to the number of unique conformers generated, number of unique ring conformations, computational speed, ability to find the global minimum and, the ability to identify conformers similar to the experimentally determined X-ray conformation after Protein Preparation Wizard treatment (X-ray_ppw_) and to the X- ray_ppw_ ring conformation.

#### Number of generated conformers and ring conformations

The number of generated conformers were extracted from the conformational search log files (.log files). The number of ring conformers generated by each method was investigated via the Redundant Conformation Elimination method implemented in MacroModel (for specialized settings see “Calculating the Number of Generated Conformers and Ring Conformations.” in supporting information). The heavy atoms in the macrocyclic ring were superimposed and redundant conformers were eliminated based on a maximum atom deviation cut-off of 0.5 Å. The Retain Mirror Image conformation option was used. The energy window for conformer selection was set to 62.8 kJ mol^−1^ (15.01 kcal mol^−1^).

#### Computational speed

To compare computational times between methods, the CPU times were extracted from the log files (.log-file).

#### Identifying the global energy minimum

The global energy minimum conformer was considered as identified if a method generated a conformer with an energy difference not greater than 1 kJ mol^−1^ compared to the lowest energy conformer found by any method for that macrocycle (here assumed to correspond to the global energy minimum). As an additional evaluation, the similarity in geometry between the global energy minimum and the lowest energy conformer within 1 kJ mol^−1^ from the global energy conformer generated by the other methods, was analyzed. Here two different RMSD values using only the heavy atoms in the macrocyclic ring and all heavy atoms, respectively, were calculated.

#### Producing a conformation similar to the X-ray_ppw_ conformation

The ability of different search methods to generate conformers similar to the experimentally determined conformation was evaluated by calculating the RMSD between the heavy atoms in the ligand X-ray structure after Protein Preparation Wizard treatment (called X-ray_ppw_) and the generated conformers using the superposition tool in Maestro.

#### Producing a conformation similar to the X-ray_ppw_ ring conformation

The ability of the different search methods to generate ring conformations similar to the experimentally determined X-ray ring conformation was evaluated by calculating the RMSD_RING_ between the heavy ring atoms in the X-ray structure after Protein Preparation Wizard treatment (X-ray_ppw_) and the generated conformers using the superposition tool in Maestro.

## Results and discussion

This study aimed to evaluate the performance of the more general and well-established conformational analysis methods MCMM and MTLMOD in comparison with the new specialized macrocycle sampling techniques MD/LLMOD and PRIME-MCS. Given the importance of macrocycle-protein modelling in drug discovery, we envisaged that a systematic study of both classical and recent specialized methods would provide guidance for other practitioners within the field. In addition to assessing the relative performance of these conformational search methods, we also wanted to address the challenge of performing conformational analysis of large macrocyclic structures with many rotatable bonds. This included studying the degree of conformational space covered in a conformational search. The energy differences between the conformers most similar to the X-ray_ppw_ conformation and the lowest energy conformation identified were also studied. However, the default settings of the general methods have not necessarily been optimized to perform well on macrocycles [32]. Therefore, using 10 macrocycles as a representative subset of the full data set, we first investigated whether small changes to the MCMM and MTLMOD methods could enhance conformational sampling of these challenging ring systems and improve the X-ray_ppw_ reproduction accuracy. Thereafter, we used the full data set with macrocycles extracted from 44 crystal structures and compared the two general methods (with and without enhanced settings) with the two more specialized methods MCS-PRIME and MD/LLMOD.

Conformational sampling can be run using many different settings. To enable fair comparison of the current work with previous studies, we opted to employ 10,000 search steps per structure, an amount that should be feasible for most modelling projects [33, 34, 41, 64]. To further align our work with the literature, an energy window of 15 kcal mol^−1^, [44] and up to 50,000 minimization steps [32] was used. For all methods except MD/LLMOD and MCMM-Exhaustive, three runs with different seeds were performed to assess how the stochastic element of the searches affected the results [44]. Finally, we ran an exhaustive conformational search using the MCMM-Enhanced settings and 1,000,000 search steps per structure to compare with the results obtained from the searches using only 10,000 steps search per structure.

It should be noted that when the MD/LLMOD method was developed it was trained on about two-thirds of the macrocycles used in this study, which could potentially bias the results [32].

### Data set selection

In general, macrocycle conformational analysis studies have used structures obtained from both the PDB and the Cambridge Structural Database (CSD) [65], with the majority of the structures retrieved from the latter. A significant difference between these databases is that reported macrocycle structures are typically crystallised either with or without protein partners in the PDB and CSD, respectively. Whilst reported conformations of macrocycles reported in both the “free” and protein-bound state can be similar, they may also diverge significantly [36]. Given our emphasis on biologically relevant macrocycles, we directed efforts towards X-ray conformations extracted from the PDB as these protein–ligand complexes can be considered as the bioactive, bound-state conformations. This allowed us to exclusively study if the conformational analysis methods could produce conformations similar to the protein-bound macrocycle conformations.

Currently, there are several macrocycle data sets publicly available. Two of the most frequently used were published by Chen and Foloppe [44] and Watts et al*.* [32]. In the present study we used the 47 protein-macrocycle complexes previously published by Alogheli et al*.* [40] Briefly, the Alogheli et al*.* data set originates from the 150 structures collected by Watts et al*.* However, all 83 structures obtained from the CSD were removed. The remaining 67 PDB structures were further filtered where structures with either a ring size below 10 atoms, an overall resolution above 2.5 Å, poor ligand resolution, uncertain stereochemistry, predominantly solvent exposed ligands, extensive ligand-ligand interactions and structures that did not overlap with the binding site produced by SiteMap [66], were removed. After this, 31 structures remained. Subsequently, using the same criteria, 16 PDB structures containing macrocycles were added, which gave 47 structures altogether. As described in the Methods section, there are three duplicate structures in the Alogheli data set (Erythromycin in 2IYF/3FRQ, Tacrolimus in 1FKJ/4NNR, and Geldanamycin in 1YET/2ESA). To avoid duplicate sampling, 2IYF, 1FKJ and, 1YET were removed from the present data set. Thus, the number of unique macrocycles in our data set is 44 and the number of unique ring system is 38.

The macrocycle ring sizes varied between 11 and 29 ring atoms (median 16), and the number of rotatable bonds ranged from 8 to 47 (median 23), see Table 1 for 2D structures and Table 2 for a summary of some characteristics of the data set.

**Table 2:** Characteristics of the Full Data set Consisting of 44 Macrocycles

Property	Average	Median	Minimum	Maximum
PDB resolution (Å)	1.88	1.88	0.95	2.50
Ring size	17	16	11	29
#Torsional angles sampled^a^	25	23	8	47
Molecular weight	571	538	280	1041
DonorHB^b^	2.5	2.0	0	9.3
AcceptHB^c^	12.0	11.2	5.3	26.9
QPlogPo/w^d^	2.7	2.8	− 2.6	6.8
PSA^e^	142	124	71	411

All descriptors were calculated using QikProp, except for ring size and the number of torsional angles sampled, which were calculated by hand. For 2XYT descriptors were calculated using Instant JChem [83]

^a^Number of torsional angles sampled during the MCMM and MTLMOD conformational searches

^b^Number of hydrogen bond donors

^c^Number of hydrogen bond acceptors

^d^Calculated octanol/water partition coefficient

^e^Polar surface area

### Generating a non-biased starting conformation

In a conformational analysis study, it is less of a challenge to generate a conformation close to the X-ray conformation if the starting conformation is geometrically similar. Therefore, to ensure a starting geometry sufficiently dissimilar from the X-ray structure, it is typical to convert the X-ray structure to a 2D SMILES string and then convert it back to a 3D structure (keeping stereochemical information). In this study we used an alternative approach where conformational ensembles generated via a 10,000 step MCMM search were generated for each macrocycle and compared to the corresponding X-ray_ppw_ conformation. The conformer with the highest atomic RMSD to the X-ray_ppw_ conformation was then chosen as the starting conformation for all subsequent conformational analysis studies. Using this approach no starting conformers had RMSD values below 1 Å to the X-ray_ppw_ conformation, see Table 3 (“starting conformer”) and Table S1 for detailed results. This should be compared with the generally accepted procedure of converting SMILES strings to 3D structures, which had four structures below the 1 Å RMSD threshold. However, it is well-known that one can obtain a high RMSD between two structures by altering only one or a few torsional angles, leaving all other geometric parameters unchanged. Therefore, to further evaluate the similarity between the starting conformers and the X-ray conformation, the torsional RMSD values were calculated.

**Table 3:** Comparing different strategies to generate non-biased starting conformers

	RMSD (Å)^a^		
Conformer	< 1 Å	1 Å–2 Å	> 2 Å
Energy minimized X-ray_ppw_ ligand	40	5	0
Starting conformer	0	7	38
SMILES conformer	4	15	26

^a^RMSD for the conformer identified with the lowest RMSD value to the X-ray_ppw_ ligand. The conformers are, dependent on their calculated RMSD values, divided into three different groups with RMSD values: below 1 Å, between 1–2 Å, and greater than 2 Å

Comparing the torsional RMSD values between the two different approaches, the median torsional RMSD were 64.3° and 68.9° for the SMILES generated conformers and the conformational ensemble generated starting conformers, respectively (Table S1). For comparison, the energy minimized X-ray_ppw_ conformations had a median torsional RMSD of 8.9°. We also compared the number of torsional angles that differed by more than 120° in comparison to the X-ray_ppw_ conformation, which were similar for the two approaches. The median number of torsional angles differing by more than 120° was five for both the starting conformer and the SMILES generated conformer. The median number of torsional angles differing by more than 60° was slightly higher for the starting conformers (16 torsional angles) compared to the SMILES conformers (14 torsional angles). Thus, no major difference was observed between the two approaches for generating a starting conformation of sufficient dissimilarity to the X-ray_ppw_ structure. Taken together, we used the most dissimilar structure based on atomic RMSD as the starting conformation in the conformational analysis studies. Overall, this conformation was more dissimilar to the X-ray_ppw_ conformation as compared to the SMILES generated conformer, since four of the SMILES generated conformers had atomic RMSD values below the 1 Å threshold. As a general note, evaluating the dissimilarity between starting and X-ray_ppw_ conformations is advisable irrespective of the generating method.

### Exploring energy minimization method and ring closure settings for MCMM and MTLMOD on a diverse subset of 10 macrocycles

#### Energy minimization method

Chen and Foloppe have shown that the settings for sampling macrocycles can be enhanced for improved search performance [44]. As it was observed that a major part of the conformational searches was spent on energy minimization of the generated conformations, the choice of minimization method was investigated using the diverse 10 macrocycle subset. The conformational search methods in MacroModel offer a wide variety of minimization methods, where the Polak-Ribiere Conjugated Gradient method (PRCG) is the default method while the truncated-Newton conjugate-gradient (TNCG) is described as a superb method for flexible structures [63]. Newton based minimization methods have also been frequently employed [30, 41, 67]. The PRCG and TNCG minimization methods were compared for the two conformational analysis methods MCMM and MTLMOD. To align all minimizations, up to 50,000 minimization steps were chosen since this is default setting for the MD/LLMOD method (the minimization terminates when the convergence criterion is met). In summary, applied on the diverse macrocycle subset, MCMM and MTLMOD ran 1.6 times and, 7.5 times faster, respectively, using TNCG instead of PRCG, see Table 4. Therefore, all minimizations with the MCMM and MTLMOD methods were run with TNCG instead of PRCG in this study.

**Table 4:** Computational times used in the conformational analysis of the ten macrocycles in the diverse subset using two different minimization methods

Conformational search method	Energy minimization method	Computational time^a^
MCMM^b^	PRCG^c^	856
MCMM^b^	TNCG^d^	544
MTLMOD^e^	PRCG^c^	4109
MTLMOD^e^	TNCG^d^	551

^a^The sum total of computational time (minutes) consumption for conformational analysis of ten macrocycles

^b^Monte Carlo Multiple Minimum

^c^Polak-Ribiere Conjugated Gradient

^d^Truncated Newton Conjugated Gradient

^e^Mixed torsional/Low-mode

#### Ring closure criterion for MCMM and MTLMOD conformational searches

The MCMM and MTLMOD search methods are implemented in MacroModel. The MTLMOD method uses either LowMode steps or MCMM steps [67]. To generate a new conformation for ring-containing compounds using the MCMM or MTLMOD methods, the ring needs to be temporarily opened, thus, a cleavage site must be identified. Using ths default settings the acceptance criteria for ring closure after torsional variation is 0.5–2.5 Å. To evaluate the performance if all opened rings are re-closed, a very wide ring closure criteria between 0 and 100 Å was investigated. Similar wide ring closure distances have been used in previous studies (e.g., 0.1–30 Å) [30]. Cleavage sites adjacent to a stereocenter can potentially present complications as reconnecting the two atoms after the torsional movement may induce inversion of the original stereocenter. Accordingly, a chirality check is used to reject conformations where this occurs. Therefore, avoiding stereocenters as ring closure atoms might increase the number of generated conformations by reducing the amount rejected due to altered stereochemistry. Applied on the 10 diverse macrocycles, the performance for both MCMM and MTLMOD when changing the ring opening width and ring-opening placement were evaluated. As expected, avoiding ring-closures adjacent to chiral centers and increasing the ring opening width provided the highest number of unique ring conformations (Table 5). This combination also generated at least one conformer within 2 Å RMSD to the X-ray_ppw_ conformation for all 10 macrocycles. We reasoned that the ability to generate many ring conformations when analysing macrocycles is of key importance and therefore these settings were used in all subsequent studies (termed MCMM-Enhanced and MTLMOD-Enhanced). A more detailed walkthrough of the modified parameters is described in section “Method Optimization Using a Diverse Subset of 10 Macrocycles” in the supporting information.

**Table 5:** Summary of conformational analysis settings and results of the 10 macrocycles in the diverse subset

Method	Ring opening	Ring closure distance (å)	No. conf^a^	No. unique ring conf^b^	CPU time (min)^c^	Global energy minimum found for no. Macrocycles^d^	Best fit conformation RMSD (Å)^e^		
							< 1 Å	1 Å–2 Å	> 2 Å
MCMM	Standard	0.5–2.5	40,507	2482	666	4	7	0	3
MCMM	Standard	0.1–5.0	40,946	5158	646	3	8	1	1
MCMM	Standard	0–100	36,034	9326	813	5	7	3	0
MCMM	Moved	0.5–2.5	41,538	2811	614	3	6	2	2
MCMM-Enhanced	Moved	0–100	38,037	9402	800	5	7	3	0
MTLMOD	Standard	0.5–2.5	29,417	4082	714	4	6	1	3
MTLMOD-Enhanced	Moved	0–100	32,489	7574	704	3	8	2	0

^a^The sum total of conformers generated

^b^The sum total of unique ring conformations generated

^c^The sum total of computational time (minutes) used for conformational analysis

^d^Number of macrocycles where the lowest energy conformer was identified or a conformer with an energy difference no greater than 1 kJ mol^−1^

^e^RMSD for the conformer identified with the lowest RMSD value to the X-ray ligand after protein preparation treatment (X-ray_ppw_). The conformers are, dependent on their RMSD values, divided into three different groups with RMSD values: below 1 Å, between 1 Å–2 Å, and greater than 2 Å

### Comparing all search methods using the full data set of 44 macrocycles

To compare the general conformational search methods (MCMM, MTLMOD) with the more specialized macrocycle-sampling methods (MD/LLMOD, PRIME-MCS) we applied these methods on all macrocycles contained in the full data set. As the MCMM-Enhanced and MTLMOD-Enhanced methods performed well for the diverse subset, these were also included in the comparison study. Methods were evaluated based on the following criteria; the ability to identify (i) unique conformers, (ii) unique macrocycle ring conformations, (iii) the global energy minimum, and (iv) the methods’ computational speed and (v) the ability to identify conformers similar to the X-ray_ppw_ conformation, and (vi) to the X-ray_ppw_ ring conformation. To evaluate how well the different conformational search methods performed and to get an approximation of the search efficiency, it would also be interesting to compare the generated conformational ensembles with the complete set of all possible conformers. However, as the number of conformers increases almost exponentially with the number of rotatable bonds, generation of such complete ensembles is difficult [64]. To at least address this challenge, the conformational search methods examined in this work were benchmarked against an exhaustive MCMM run using 1,000,000 search steps. The MCMM method was used in this study because it is an efficient method for generating conformers and the most efficient in generating ring conformations. MCMM, MCMM-Enhanced, MTLMOD, MTLMOD-Enhanced and PRIME-MCS were run three times independently using different seeds. The settings used for the different methods are summarized in Table 6 and all results are summarized in Table 7. For those methods where 3 different seeds were used, the Max, Min and Average results for each method are presented.

**Table 6:** Summary of the conformational analysis settings for the evaluated methods and literature protocols

Method	Number of search steps	Energy window (kcal mol^−1^)	Torsion sampling option^a^	Elimination of redundant conformations^b^	Ring closure distance (Å)^c^	Placement of ring opening^d^	Energy minimization method^e^	Maximum energy minimization iterations	Energy minimization threshold (kJ Å^−1^ mol^−1^)	Force field	Solvent
MCMM default^f^	1000	5.02	Intermediate	AD 0.5 Å	0.5–2.5	Standard	PRCG	2500	0.05	OPLS-3	water
MTLMOD default^f^	1000	5.02	Intermediate	AD 0.5 Å	0.5–2.5	Standard	PRCG	2500	0.05	OPLS-3	water
MD/LLMOD default^f^	5000 MD, 5000 LLMOD	10	Enhanced	RMSD 0.75 Å	NA^g^	NA^g^	NAV^h^	50,000	0.01	OPLS-3	water
MD/LLMOD	5000 MD, 5000 LLMOD	15.01	Extended	RMSD 0.75 Å	NA^g^	NA^g^	NAV^h^	50,000	0.01	OPLS-3	water
MCMM	10,000	15.01	Extended	AD 0.5 Å	0.5–2.5	Standard	TNCG	50,000	0.05	OPLS-3	water
MCMM-Enhanced	10,000	15.01	Extended	AD 0.5 Å	0 – 100	Stereocenters avoided	TNCG	50,000	0.05	OPLS-3	water
MCMM-Exhaustive	1,000,000	15.01	Extended	AD 0.5 Å	0–100	Stereocenters avoided	TNCG	50,000	0.05	OPLS-3	water
MTLMOD	10,000	15.01	Extended	AD 0.5 Å	0.5–2.5	Standard	TNCG	50,000	0.05	OPLS-3	water
MTLMOD-Enhanced	10,000	15.01	Extended	AD 0.5 Å	0–100	Stereocenters avoided	TNCG	50,000	0.05	OPLS-3	water
PRIME-MCS	Spinroot 10^i^	100	Peptide bonds	Torsional fingerprint	NA^g^	NA^g^	TNCG	Chain minimization^j^	0.04	OPLS-2005	vacuum
CF-MTLMOD^k^	10,000 (400 RotStep)^l^	15	Intermediate	RMSD 0.25 Å	0.5–2.5	Standard	PRCG	3000	0.05	OPLS-2005	water
CF-MD/LLMOD^k^	5000 MD, 5000 LLMOD	15	Enhanced	RMSD 0.25 Å	NA^g^	NA^g^	NAV^h^	50,000	0.01	OPLS-2005	water
CF-LowModeMD MOE^k^	10,000	15	NA^g^	RMSD 0.25 Å	NA^g^	NA^g^	NAV^h^	500	0.021	MMFF94x	water

^a^Intermediate—Sample C–N and C–O single bonds other than in standard amides and esters; Enhanced—Sample all C–N and C–O single bonds; Extended—Sample all C–N and C–O single bonds and C = N and N = N double bonds. Sampling of peptide bonds are allowed (“peptide bonds”)

^b^Atom deviation (AD): A conformation is unique if one (or several) of the defined atoms deviates more than specified, from the compared conformations after superposition. Root Mean Square Deviation (RMSD): A conformation is unique if the RMSD value between two conformations exceeds the specified value. Torsional Fingerprint: Two conformations are considered redundant if they have identical torsional fingerprints

^c^Re-close ring system if the ring closure atoms are within the defined distance range

^d^Placement of the macrocyclic ring opening bond could be adjacent to a stereocenter using the automatic setup (standard)

^e^Energy minimization method. Polak-Ribiere Conjugated Gradient (PRCG). Truncated Newton Conjugated Gradient (TNCG)

^f^Default refers to the predefined values in Schrödinger

^g^Not Applicable

^h^Not Available

^i^Spinroot 1, and 10 generating up to 100 and 1000 conformations, respectively

^j^Chain energy minimization, starts with a conjugate gradient followed by a Truncated Newton minimization

^k^Enhanced settings presented by I-Chen and Foloppe

^l^Limits the total number of search steps as a function of the number of rotatable bond. Only active if multiple ligands are sampled simultaneously

**Table 7:** Summary of conformational analysis results for the full data set of 44 macrocycles

Method	No. conf^a^	No. unique ring conf^b^	Computational time (min)^c^	Global energy minimum found for no. macrocycles^d^	Best fit conformation RMSD (Å)^e^			RMSD_RING_ (Å)^f^		
					< 1 Å	1 Å–2 Å	> 2 Å	< 0.5 Å	0.5 Å–1 Å	> 1 Å
Energy minimized X-ray_ppw_ ligand	NA^g^	NA^g^	NA^g^	NA^g^	40	5	0	45	1	0
Starting conformer	NA^g^	NA^g^	NA^g^	NA^g^	0	7	38	3	19	24
SMILES conformer	NA^g^	NA^g^	NA^g^	NA^g^	4	15	26	5	25	16
MCMM^h^										
Average	155,296	18,950	3740	48% (63/132)	37	6	2	38	8	0
Max	159,973	20,274	4138	NA^g^	42	0	3	42	2	2
Min	150,558	17,840	3340	NA^g^	32	13	0	35	11	0
MTLMOD^h^										
Average	117,490	23,367	4125	48% (64/132)	35	8	2	39	6	1
Max	121,147	25,015	4468	NA^g^	39	5	1	42	1	3
Min	113,512	21,804	3806	NA^g^	31	10	4	37	9	0
MCMM-Enhanced^h^										
Average	149,831	49,324	4642	52% (68/132)	40	5	0	44	2	0
Max	155,304	52,181	5022	NA^g^	41	4	0	45	1	0
Min	144,227	46,906	4325	NA^g^	35	10	0	40	6	0
MTLMOD-Enhanced^h^										
Average	134,396	41,040	4182	50% (66/132)	36	9	0	45	1	0
Max	137,988	42,589	4515	NA^g^	41	3	1	46	0	0
Min	130,954	39,443	3932	NA^g^	32	13	0	41	5	0
MD/LLMOD^i^										
Average	45,917	19,189	4163	55% (24/44)	31	11	3	38	8	0
Max	NA	NA	NA	NA^g^	NA	NA	NA	NA	NA	NA
Min	NA	NA	NA	NA^g^	NA	NA	NA	NA	NA	NA
PRIME-MCS^h^										
Average	31,118	24,953	9791	NA^g^	24	19	2	35	11	0
Max	31,286	25,122	9804	NA^g^	24	19	2	37	9	0
Min	30,952	24,795	9779	NA^g^	22	21	2	34	12	0
MCMM-Exhaustive	7,528,356	967,844	925,026	45	44	1	0	46	0	0

^a^The sum total of conformers generated

^b^The sum total of unique ring conformations

^c^The sum total of computational time (minutes) used for conformational analysis

^d^Number of macrocycles where the lowest energy conformer was identified or a conformer with an energy difference not greater than 1 kJ mol^−1^ and an RMSD below 0.1 Å to the global energy conformer using all heavy atoms

^e^RMSD for the conformer identified with the lowest RMSD value to the X-ray ligand after protein preparation treatment (X-ray_ppw_). The conformers are, dependent on their RMSD values, divided into three different groups with RMSD values: below 1 Å, between 1–2 Å, and greater than 2 Å

^f^RMSD_RING_ for the conformer identified with the lowest RMSD_RING_ value to the heavy ring atoms in the X-ray ligand after protein preparation treatment. The conformers are, dependent on their RMSD values, divided into three different groups with RMSD values: below 0.5 Å, between 0.5–1 Å, and greater than 1 Å. 3BXR consist of two macrocyclic rings, therefore 46 (instead of 45) RMSD_RING_ values are presented

^g^Not Applicable

^h^Run three time using different seeds.

^i^MD/LLMOD was run one time

PRIME-MCS employs, in comparison with other methods that use 15 kcal mol^−1^, a much wider energy window of 100 kcal mol^−1^ for saving conformers, which may result in a larger number of conformers generated. Furthermore, PRIME-MCS minimizations are performed in vacuum as compared to the GB/SA water solvation model that is used by the other methods. Therefore, whilst the results of PRIME-MCS are not directly comparable with the MCMM, MTLMOD and MD/LLMOD methods, they are still included as a comparison in all results except in the search for the global energy minimum.

#### Total number of conformers generated

For each of the three runs using different seeds, the sum of all conformers identified for all 44 macrocycles was calculated for each search method. Since all methods except MD/LLMOD were run three times, the average number of conformers per run will be presented to allow a comparison between the methods. This number was calculated as the sum of identified conformers for each method divided by the number of runs that were made for that method. Across all search methods, MCMM generated the highest average number of conformers over all 44 macrocycles (155,296), see Table 7 and Table S9. MCMM-Enhanced identified 149,831 conformers on average followed by MTLMOD-Enhanced (134,396), MTLMOD (117,490), MD/LLMOD (45,917), and PRIME-MCS (31,118).

In the 10,000 step runs, the variation in number of identified conformations using the three different seeds did not vary considerably within the different methods for each macrocycle. The largest observed difference was for MCMM-Enhanced with a variation of 7% between the highest and lowest number of generated conformations (see max/min in Table 7). For example, for 1BXO, which contains 24 rotatable bonds, MCMM-Enhanced generated 6141/5345/5963 conformations out of 10,000 possible for each run.

With 155,296 identified conformers, MCMM produces a new conformer within the given energy window approximately every third iteration (440,000 possible conformers if every Monte Carlo step would generate a new conformer). Using 100 times as many search steps, the MCMM-Exhaustive search identified about 48 times more conformers in total (7,528,356), as compared to the shorter MCMM protocol (discussed further in section “Conformational coverage—are we reaching convergence”) However, the MCMM-Exhaustive searches were intended to serve as a benchmark in this study and generating large conformational ensembles can be problematic in terms of disk space, data handling and, downstream processing such as visual inspection, docking, pharmacophore modelling and quantum mechanical optimizations, etc.

#### Number of unique ring conformations generated

As not all conformational sampling software support macrocyclic ring sampling and since ring sampling in itself is not always easily performed, [36] we evaluated the different methods’ ability to generate unique ring conformations. This was defined as the sum of identified ring conformations for each method divided by number of runs that were made for that method. Whilst one could hypothesize that generating more conformers would also provide more ring conformations, the sum of all ring conformations identified by each method did not parallel the total number of generated conformers for the full data set. Instead, MCMM-Enhanced produced the largest number of ring conformations (49,324) followed by MTLMOD-Enhanced (41,040), PRIME-MCS (24,953), MTLMOD (23,367), MD/LLMOD (19,189) and MCMM (18,950) (Table 7 and Table S10). Thus, running MCMM and MTLMOD using adjusted settings regarding the ring opening bond (the enhanced settings) increased the number of generated ring conformations drastically. The MCMM-Exhaustive searches generated 967,844 ring conformations in total showing that with increased sampling more ring conformations could be found.

#### Identifying the global energy minimum

The ability of the methods to identify the global energy minimum was also evaluated. As minimization uses a GB/SA solvation model in all methods except for PRIME-MCS (vacuum), this method was not evaluated in this section. As MCMM-Exhaustive (1,000,000 search steps) identified the lowest energy conformer for all macrocycles, this was considered as the global minimum. For the other methods, the global minimum energy was considered identified if a conformer within 1 kJ mol^−1^ of the global energy minima was generated. We also set out to investigate if these conformers were geometrically identical to the global energy minima conformer. This was determined by first analyzing whether the global energy ring conformation was identified and, secondly, whether the whole conformer was identified. The global energy conformer and ring conformation were considered geometrically identical if the RMSD between the two conformers were below 0.1 Å RMSD when using either all heavy atoms present or just those of the macrocyclic ring, respectively. The mirror-image conformers of the global energy minimum were considered identical in this analysis.

To compare the ability of different methods to identify the global energy minimum using 10,000 search steps, the percentage of runs in which these conformations were identified was calculated. By only considering the energy, MD/LLMOD identified the global energy minima in 64% of the conformational searches (28/44), whereas MTLMOD-Enhanced and MCMM-Enhanced identified a conformer within 1 kJ mol^−1^ from the global energy minimum for 60% (79/132) and 59% (78/132) of the runs, respectively, see Table 7 and Table S11. MCMM and MTLMOD found the global energy minimum for and 57% (75/132), 56% (74/132) of the runs, respectively. Consequently, when using the enhanced settings, both MCMM and MTLMOD performed slightly better. Since the success rate of finding a conformer within 1 kJ mol^−1^ from global minimum ranged between 56 and 64%, this suggests that the 10,000 search steps might not be adequate for finding the global minimum for macrocycles. However, considering the number of rotatable bonds a macrocycle may have and, consequently, the large number of conformers that may exist, the probability of generating the global energy minimum should be rather low. Thus, the low success rates of generating the global energy minimum are not surprising.

By requiring that the ring conformation should be identical to that of the global energy minimum, MD/LLMOD instead identified the global energy minima in 61% (27/44) of the conformational searches. This was followed by MCMM-Enhanced 57% (75/132), MTLMOD-Enhanced 55% (73/132), MTLMOD 54% (71/132) and, MCMM 53% (70/132). Using the strictest definition were the whole (all heavy atoms) global energy minima conformer needs to be identical, MD/LLMOD identified the global energy minima in 55% (24/44) of the conformational searches, followed by MCMM-Enhanced 52% (68/132), MTLMOD-Enhanced 50% (66/132), MTLMOD 48% (64/132) and, MCMM 48% (63/132). In summary, MD/LLMOD generated the global energy minima most frequently of the evaluated methods across all three definitions of the global energy minima.

**Fig. 2:**
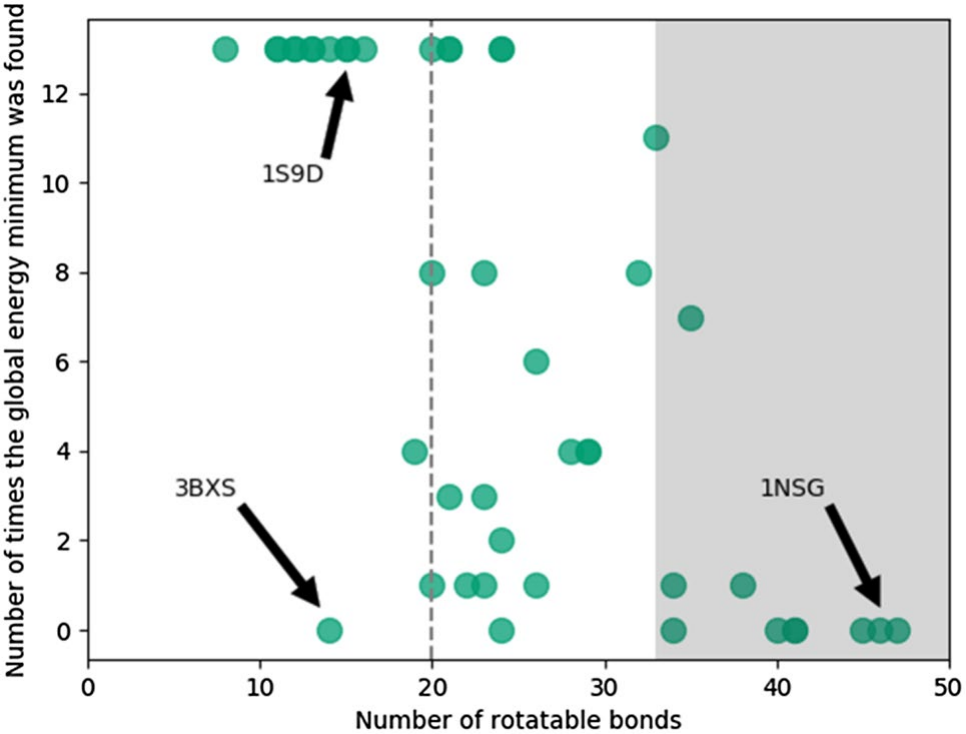
Relationship between rotatable bonds defined as the number of torsion angles sampled per macrocycle and the number of times the global energy minima were identified using the different methods (13 runs per macrocycle using 6 different methods)

The challenge of identifying the global energy minimum for a given macrocycle can be estimated by the number of times it is found over 13 different runs (3 MCMM runs, 3 MCMM-Enhanced runs, 3 MTLMOD runs, 3 MTLMOD-Enhanced runs and 1 MD/LLMOD run) [44]. For each macrocycle, the relationship between the number of times the methods found the global energy minimum (using the all heavy atoms definition) and the number of rotatable bonds is shown in Fig. 2 below. For example, all 13 methods identified the global energy minimum for 1S9D (15 rotatable bonds), while none of the methods found the global energy minima for 1NSG (46 rotatable bonds). As expected, the methods are more successful for identifying the global energy minimum for less flexible macrocycles (less than 20 rotatable bonds, dotted line in Fig. 2). In contrast, this is more challenging for flexible macrocycles with more than 33 rotatable bonds (grey area in Fig. 2). This suggests that if the aim is to generate the global energy minimum for a macrocycle with many rotatable bonds, more extensive conformational sampling than 10 000 search steps is required. For nine macrocycles, none of the methods except MCMM-Exhaustive were able to identify the global energy minimum (1FKD, 1FKI, 1NMK, 1NSG, 1TPS, 2ASP, 2DG4, 3BXS and 4NNR). These compounds contain some of the largest ring structures in the data set except 3BXS. In 3BXS none of the methods identified the correct valine side-chain orientation.

#### Computational speed

The computational speed per method is given as the average CPU-time to sample the 44 macrocycles (total CPU time/number of seeds). The fastest method overall, was MCMM (3740 min) followed by MTLMOD (4125 min), MD/LLMOD (4163) MTLMOD-Enhanced (4182 min), MCMM-Enhanced (4642 min) and PRIME-MCS (9791 min), see Table 7 and Table S12. PRIME-MCS (9791 min) was thereby roughly twice as slow in comparison to the second slowest method MCMM-Enhanced (5345 min). MCMM-Exhaustive ran for 925,026 min, *i.e*. almost 2 years, in total (median 17,412 min, approximately 12 days per macrocycle). Other conformational sampling methodologies requiring up to 7 days on a 100 CPU cluster have been published [37]. However, this amount of conformational sampling is rarely described. Thus, using MCMM-Exhaustive is probably unreasonably lengthy for most modelling projects in the field of drug discovery.

#### Generating a conformation similar to the X-ray_ppw_ conformation

The ability to generate conformers similar to the bioactive conformation in the X-ray structure (best-fit conformation in Table 7) was analysed by calculating the RMSD between all conformers for each macrocycle and the X-ray ligand after Protein Preparation Wizard treatment (the X-ray_ppw_ conformation). Structures downloaded from the PDB are interpretations of electron densities and therefore models that may contain errors, especially in the ligand structures [68-71]. Therefore, we allowed for “adjusting” distorted bond angles and lengths, etc., in protein–ligand complexes and thereby aligning the structures to the force field. Thus, we used the X-ray_ppw_ conformation as the X-ray structure in all comparisons. When available, the fit to the electron density was evaluated for the protein-macrocycle complexes after the restrained energy minimization. The conformer most similar to the X-ray_ppw_ conformation was grouped in one of the following categories based on the RMSD value; below 1 Å, between 1 Å–2 Å and greater than 2 Å. All non-hydrogen atoms were considered for the RMSD calculations. Furthermore, since one of the complexes (3BXS) contains the macrocycle bound to two separate binding sites with different conformation, 45 instead of 44 macrocycles extracted from protein-macrocycle complexes were evaluated in this section. The median RMSD value between the X-ray ligand structure before and after protein preparation wizard treatment was 0.19 Å. For methods run in triplicate, the mean value is presented.

Furthermore, to explore the local minimum closest to the X-ray_ppw_ conformation we energy minimized the X-ray_ppw_ structure and compared it with the X-ray_ppw_ conformation (“energy minimized X-ray_ppw_ ligand” in Table 7). 40 out of the 45 macrocycles had a local minimum with RMSD values below 1 Å when superimposed on the X-ray_ppw_ structure, and five energy minimized X-ray_ppw_ conformers had RMSD values between 1 and 2 Å, see also Table S13.

**Fig. 3:**
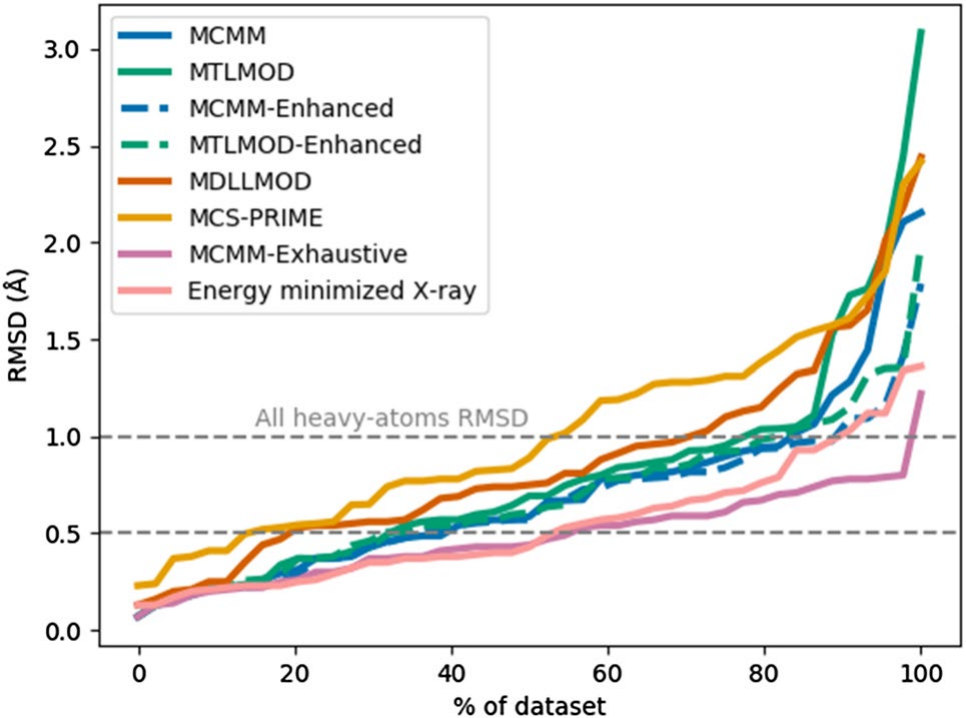
All heavy-atoms RMSD. The mean RMSD value was used for those methods that were run more than one time. The cumulative performance describing how successful the methods are at generating a conformer close to the X-ray_ppw_ conformation is shown. The performance is benchmarked against the energy minimized X-ray_ppw_ conformations (shown by the pink line near the bottom) and an exhaustive MCMM run (1,000,000 search steps shown by the purple line at the bottom)

The different methods’ ability to generate conformers similar to the X-ray_ppw_ conformation can be seen in Table 7 (“best fit conformation”), Table S13 and Table S14. Analysing the results below 1 Å RMSD, the MCMM-Exhaustive searches were able to identify such conformers for 44 out of 45 macrocycles. The RMSD for 1TPS was 1.22 Å and this macrocycle contained the highest number of rotatable bonds (47), which could be a reason for this. The second best search method was MCMM-Enhanced (40 of 45 macrocycles), followed by MTLMOD-Enhanced (36 out of 45 macrocycles). The more specialized methods MD/LLMOD and PRIME-MCS searches generated a conformer below 1 Å RMSD for 31 and 24 macrocycles, respectively. The results from the MCMM-Exhaustive search imply that conformers very close to the X-ray_ppw_ conformations can be generated if the sampling is sufficiently increased. Surprisingly, when comparing the MCMM-Enhanced and the MTLMOD-Enhanced searches with the MCMM-Exhaustive search the results are not dramatically different. Thus, 10,000 search steps seems to be adequate for generating a conformer close to the X-ray_ppw_ conformation for the enhanced methods. Overall, the general methods seemed more efficient at generating conformers close to the X-ray_ppw_ conformations in comparison to the more specialized methods. A visual overview of how the different methods performed is depicted in Fig. 3.

#### Generating the X-ray_ppw_ ring conformation

Most of the published macrocycle sampling studies have focused on the RMSD to the macrocyclic ring atoms as found in the X-ray. Thus, as a final comparison, we wanted to evaluate if the methods examined here could identify a ring conformation similar to the X-ray_ppw_ ring conformation (RMSD_RING_). These results are summarized in Table 7 and are presented in greater detail in Tables S15 and S16. For 3BXR, which consists of two macrocyclic rings, an RMSD_RING_ value was calculated for each ring separately. Therefore, 46 RMSD values instead of 45 will be presented in this section. For methods run in triplicates, the mean value is presented.

**Fig. 4:**
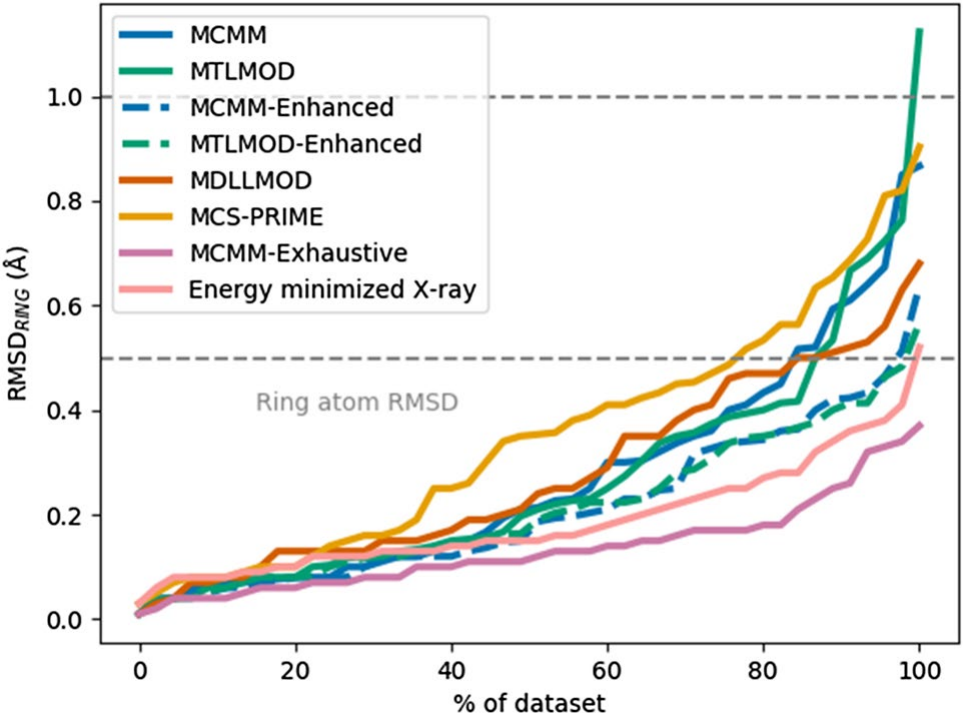
Ring atoms RMSD. The mean RMSD value was used for those methods that were run more than one time. The cumulative performance describing how successful the methods are at generating a conformer close to the X-ray_ppw_ conformation is shown. The performance is benchmarked against the energy minimized X-ray_ppw_ conformations (shown by the pink line near the bottom) and an exhaustive MCMM run (1,000,000 search steps shown by the purple line at the bottom)

Ideally, a conformational search method should be able to identify a conformer below 0.5 Å RMSD_RING_ (a commonly used threshold [33, 36, 38]). Analysing the results of the benchmarking methods MCMM-Exhaustive and the energy minimized X-ray_ppw_ structures, these methods were able to identify a conformer below 0.5 Å RMSD_RING_ in 46 and 45 cases out of the 46 macrocyclic rings, respectively. Analysing the results obtained with the other methods, they all had a median RMSD_RING_ below 0.5 Å. However, MCMM-Enhanced and MTLMOD-Enhanced most accurately regenerated the macrocyclic ring conformation and both methods identified a conformer below 0.5 Å RMSD_RING_ in 44 out of the 45 cases. For comparison, the third best method MTLMOD identified such a conformer for 39 out of the 46 macrocyclic rings. Compared to the other methods in Fig. 4, MCMM-Enhanced and MTLMOD-Enhanced seem to be more efficient at generating a ring conformations close to the X-ray_ppw_ ring conformation.

### Comparing the results with prior work in the field

Several recent publications have studied the conformational preference of macrocycles and presented new conformational sampling methods. However, a direct comparison of these methods with those presented in this study is difficult since the datasets differ and the energies, number of conformers and ring conformations, may change significantly depending on, for example, what force field has been used. Another challenge with such comparisons is that small changes in programme settings can change the internal rankings between the methods. Despite this, we compared the ability of the methods presented in each paper to reproduce the X-ray structure (X-ray accuracy). Specifically, we analyzed the methods ability to re-generate the X-ray conformation with respect to all heavy-atoms RMSD, as well as the backbone (ring) atoms RMSD_RING_ (see Table 8). As it is not meaningful to directly compare the results between different studies, we only looked at the internal order of the different methods in each paper with respect to RMSD values. The comparison starts with the well-cited work from Chen and Foloppe [44] where MD/LLMOD and MTLMOD, among other methods were evaluated, followed by an analysis of the publications where the MD/LLMOD [32] and PRIME-MCS [33] methods were published. Finally, we included the publications that introduces the new methods BRIKARD [35], PLOP [36], and ForceGen [34]. In all the publications above, with the exception of ForceGen, the MD/LLMOD method has been included, which allows it to serve as a reference method in the current analysis (the results of MD/LLMOD are shown in bold in Table 8).

**Table 8:** Summary of the X-ray accuracy reported in the literature

Method	Authors	Data set (total no. structures/PDB structures)	% Below 1 Å RMSD^a^	Median RMSD (Å)^b^	Median RMSD_RING_ (Å)^c^
MD/LLMOD	Chen and Foloppe [44]	Chen and Foloppe (30/30)	**77**	**NA**^d^	**NA**^d^
CF-MTLMOD^e^			79	NA^d^	NA^d^
MOE LowModeMD			72	NA^d^	NA^d^
Stochastic search			53	NA^d^	NA^d^
MD/LLMOD	Watts et al. [32]	Watts et al. (150/67)	**NA**^d^	**1.14**	**NA**^d^
MD/LLMOD	Sindhikara et al. [33]	Watts et al. (208/60) 60 PDB structures from Watts et al.	**NA**^d^	**1.10** (PDB)	**0.38** (PDB)
PRIME-MCS			NA^d^	1.49 (PDB)	0.40 (PDB)
MOE LowModeMD			NA^d^	1.69 (PDB)	0.41 (PDB)
Molecular dynamics simulation (24 ns)			NA^d^	1.89 (PDB)	0.56 (PDB)
BRIKARD	Coutsias et al. [35]	Coutsias et al. (67/39)	NA^d^	NA^d^	0.47 (all), 0.42 (PDB)
CF-MD/LLMOD^e^			NA^d^	NA^d^	0.54 (all), 0.47 (PDB)
MD/LLMOD			**NA**^d^	**NA**^d^	**0.63** (all), **0.49** (PDB)
CF-LowModeMD^e^			NA^d^	NA^d^	0.64 (all), 0.54 (PDB)
PLOP	Wang et al. [36]	Wang et al. (37/12)	NA^d^	NA^d^	0.25 (70% below 0.5 Å)
MD/LLMOD			**NA**^d^	**NA**^d^	NA^d^**(64% below 0.5 Å)**
CF-MTLMOD^e^	Cleves and Jain [34]	Chen and Foloppe (30/30)	NA^d^	NA^d^	NA^d^
CF-LowModeMD^e^			NA^d^	NA^d^	NA^d^
ForceGen			NA^d^	NA^d^	NA^d^
MCMM	Current work 2019	Alogheli and Watts et al. (44/44) 31 PDB structures from Watts el al.	78	0.58	0.16
MCMM-Enhanced			84	0.58	0.16
MTLMOD-Enhanced			89	0.59	0.17
MTLMOD			78	0.77	0.18
MD/LLMOD			**69**	**0.78**	**0.20**
PRIME-MCS spinroot 30			51	0.98	0.27

In all the publications above (exception of ForceGen), the MD/LLMOD method (shown in bold) has been included, which allows it to serve as a reference method

^a^Percent of macrocycles in the data set that the methods successfully generated a conformer below 1 Å RMSD to the X-ray conformation using all heavy atoms

^b^Median RMSD using all heavy atoms

^c^Median RMSD using all only the heavy atoms in the macrocyclic ring

^d^Not Applicable

^e^Optimized settings presented by Chen and Foloppe

Chen and Foloppe evaluated several different settings on a series of conformational sampling methods with the aim of optimizing search efficiency. The optimal settings derived are presented with the prefix “CF-” below and in Table 6. Unfortunately, not all heavy-atoms RMSD values were reported in this study but instead the number of X-ray structures that were reproduced within an RMSD of 1 Å. The CF-MTLMOD method was the most accurate in generating conformers below 1 Å RMSD (79%) followed by CF-MD/LLMOD (77%), MOE LowModeMD (72%), and MOE stochastic search (53%) [44].

Watts et al*.* presented the MD/LLMOD method and a macrocycle data set consisting of 67 PDB structures (150 structures in total) [32]. This data set, as well as the MD/LLMOD method, has been used in several other studies. Applied on the 67 PDB structures, Watts et al*.* reported a median heavy-atom RMSD of 1.14 Å (values reported in supporting information in ref [32]) compared to the X-ray structure).

Sindhikara et al*.* used 60 out of the 67 macrocycles in the Watts et al*.* data set and included the MD/LLMOD method, a molecular dynamics simulation method (simulations were run for 24 ns), and MOE LowModeMD [31] as reference methods when they presented and evaluated the PRIME-MCS method [33]. Applied on those 60 PDB structures, Sindhikara et al*.* reported the median all heavy-atom RMSD values to the X-ray structure to be the lowest for MD/LLMOD (1.10 Å), followed by PRIME-MCS (1.49 Å), MOE LowModeMD (1.69 Å), and molecular dynamics (1.89 Å) (values reported in supporting information in ref [33]). The median RMSD_RING_ values followed a pattern analogues to the all heavy-atom RMSD values. The lowest median RMSD_RING_ value was obtained for MD/LLMOD (0.38 Å) followed by PRIME-MCS (0.40 Å), MOE LowModeMD (0.41 Å), and molecular dynamics (0.56 Å) (values reported in supporting information in ref [33]). Thus, in both cases, the MD/LLMOD method had the best accuracy for reproducing the X-ray conformation.

In a study by Coutsias et al*.,* a new method called BRIKARD was presented [35]*.* To evaluate BRIKARD, Coutsias et al*.* collected a data set of 67 structures, of which 39 originated from the PDB. BRIKARD was benchmarked against MD/LLMOD, as well as the optimized methods CF-MD/LLMOD and CF-LowModeMD from the work of Chen and Foloppe (see Table 6 for settings). Using all 67 structures, BRIKARD had a median RMSD_RING_ value of 0.47 Å followed by CF-MD/LLMOD (0.54 Å), MD/LLMOD (0.63 Å) and CF-LowModeMD (0.64 Å) (values calculated from supporting information in ref [35]). Thus, BRIKARD seems to reproduce the ring conformation slightly better than MD/LLMOD. However, when comparing the X-ray ring accuracy for the PDB structures only, the differences between the methods were smaller. For the 39 structures originating from the PDB, the median RMSD_RING_ was 0.42 Å for BRIKARD, followed by CF-MD/LLMOD (0.47 Å), MD/LLMOD (0.49 Å), and CF-LowModeMD (0.54 Å) (median values calculated from supporting information in ref [35]).

Wang et al*.* developed the PLOP method based on 37 macrocycles originating from both the PDB and CSD [36]. In their study, MD/LLMOD and PLOP were compared based on how well the backbone (ring atoms) were reproduced. The optimized protocol of PLOP was able to reproduce the crystal structure within 0.50 Å RMSD_RING_ for 31 out of 37 macrocycles with a median RMSD_RING_ value of 0.25 Å. Wang et al. concluded that the performance of PLOP was not statistically different compared to MD/LLMOD.

Cleves and Jain compared the performance of ForceGen, CF-LowModeMD and CF-MTLMOD using the Chen and Foloppe data set (30 PDB structures) [34]. Applied on those macrocycles, CF-LowModeMD showed equivalent reproduction of the X-ray conformation (all heavy-atoms) compared to ForceGen, whereas CF-MTLMOD showed marginally better results compared to ForceGen.

To summarize, as shown from the studies above, the specialized macrocycle sampling method MD/LLMOD is able to reproduce the X-ray structures accurately, generating better or comparable results to other methods in prior publications. Interestingly, we have shown that by using slightly tweaked versions of the general methods, such as MCMM-Enhanced and MTLMOD-Enhanced, X-ray_ppw_ accuracy results comparable to, or even better than, MD/LLMOD may be obtained at least for the data set and parameters used in this study. Therefore, to further explore the general methods abilities, including MCMM-Enhanced and MTLMOD-Enhanced in future method comparison studies could be of interest.

### Conformational coverage—are we reaching convergence

The absolute degree of conformational space covered in a conformational search is often difficult to describe [72, 73]. In the literature, parameters such as the number of conformations identified, the number of times the lowest energy conformation is visited, the range of compactness/extendedness covered by the conformations as described by the radius of gyration, and the number of visited 3D pharmacophore points have been considered [31, 44]. Full conformational coverage can also be defined as when all possible conformers within a specified energy window have been found. As macrocycles are said to be conformationally restricted, we aimed to explore the conformational space in a more exhaustive way than is typical.

**Fig. 5:**
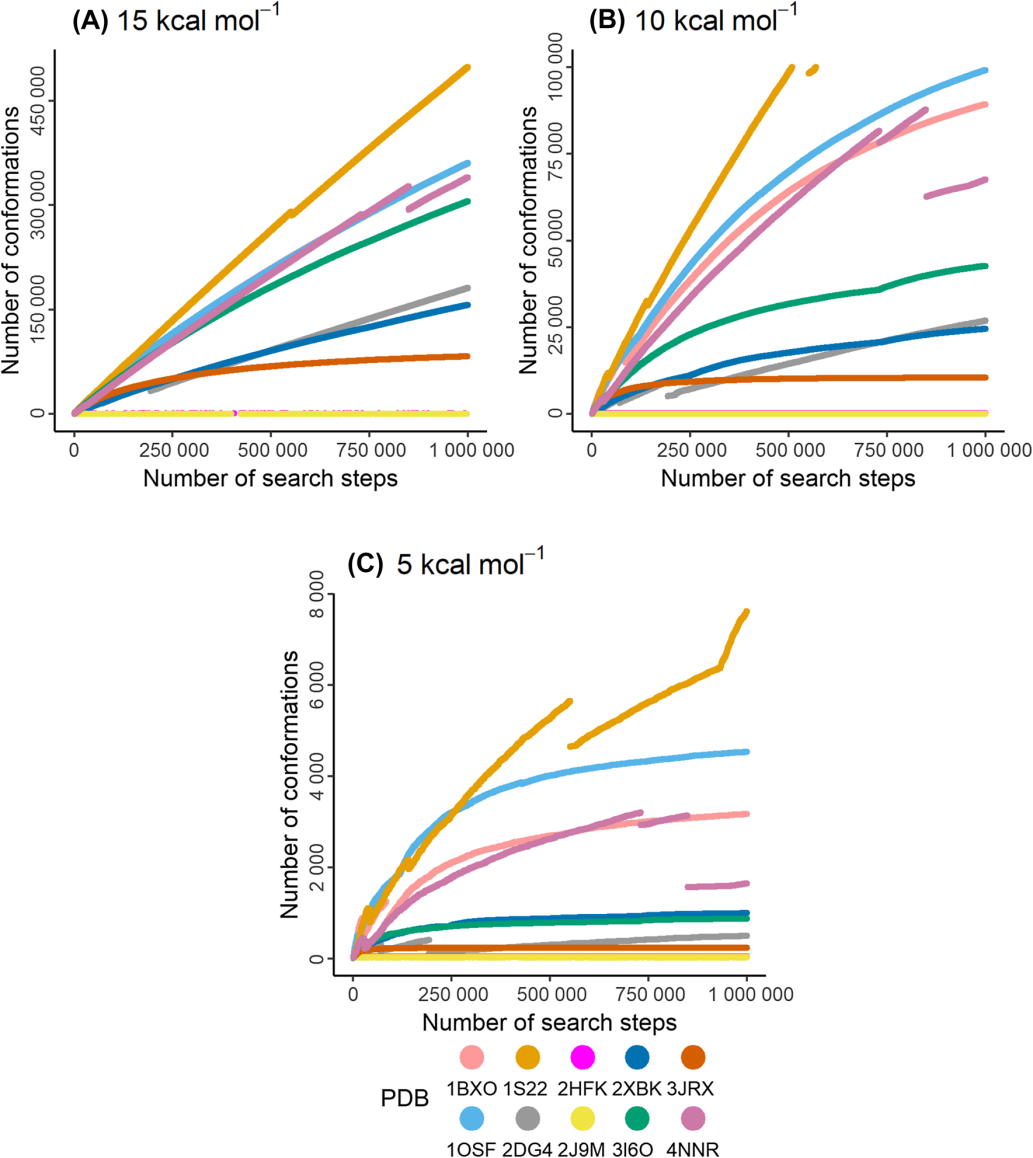
Number of generated conformers for the ten macrocycles in the diverse subset within: **a** 15 kcal mol^−1^; **b** 10 kcal mol^−1^; and **c** 5 kcal mol^−1^ from the lowest energy conformer using 1,000,000 search steps in total. The discontinuities in the lines are due to elimination of high energy conformers when a new “global energy minimum” is generated during the search. The line for 1S22 in plot (B) do not reach 1 million steps because only up to 100,000 conformers within 10 kcal mol^−1^ are registered in the .log-file

Since the shape of the energy hypersurface is force field dependent, the number of possible low-energy conformers varies between the force fields. Quantum mechanical methods would also most likely change the number of possible low-energy conformers. However, since most drug design projects are carried out in a molecular mechanics force field environment, we were interested to explore how many conformers that energy hypersurface contains. Therefore, we ran the MCMM-Enhanced search with 1,000,000 search steps (MCMM-Exhaustive) for the full data set (44 macrocycles). The results were visualized by plotting the number of search steps against the number of conformations generated within 15 kcal mol^−1^ from the global energy minimum (Fig. 5a depicts the 10 examples, chosen to represent the structurally diverse range of macrocycles). Our results show that the number of conformers generated increases steadily for all macrocycles, with the exception of 3JRX and 2HFK/2J9M, which are on top of each other. These three macrocycles have relatively small macrocyclic ring systems (12, 14 and 15, respectively) and are not extensively substituted. As these macrocycles have the smallest number of torsional angles they are therefore, expected to have fewer conformations, for example, in comparison with 1S22. This macrocycle contains a much larger ring system, substituted with a large flexible side-chain thereby increasing the degrees or torsional freedom. Thus, after 1,000,000 search steps, full conformational coverage has, as expected, not been reached for the majority of the ten displayed macrocycles. For the full data set, MCMM produced 155,846 conformers whereas MCMM-Exhaustive identified 7,528,356, corresponding to roughly 48 times more conformers than MCMM.

As expected, when examining the number of generated conformations within a narrower energy window (e.g. 10 and 5 kcal mol^−1^, in Fig. 5b and c, respectively) the rate of conformer generation decreases for most of the macrocycles. As seen in Fig. 5b, for six out of ten macrocycles (2HFK, 2J9M, 2DG4, 2XBK, 3I6O and, 3JRX) the rate of conformer generation decreases. Within 5 kcal mol^−1^, all but one (1S22) of the ten conformational searches asymptotically approaches full coverage (Fig. 5c).

Looking further at the energy distribution for all conformers of the 10 macrocycles, the vast majority of the conformers have a relative energy above 10 kcal mol^−1^. For a more thorough discussion about the energy distribution, see section “Conformational coverage and conformational energy distribution.” in the supporting information.

### Energy window for sampling macrocycles

Numerous studies have been performed to understand the conformational energy cost when a ligand binds to its target. Some argue that the acceptable conformational energy penalty is relatively low (below 3 kcal mol^−1^ [74, 75], mostly below 5 kcal mol^−1^ [76], between 4 and 6 kcal mol^−1^ [77], and mostly below 6 kcal mol^−1^ [78]) However, energies, above 9 kcal mol^−1^ [76], around 15.9 ± 11.5 kcal mol^−1^ [79], between 0 – 25 kcal mol^−1^ [80], and even up to 27 kcal mol^−1^ [81], have been suggested as feasible for protein-bound ligands. For conformational sampling of macrocycles, Chen and Foloppe noticed an improved reproduction of the X-ray conformation for MTLMOD using an increased energy window of up to 15 kcal mol^−1^ [44]. Also, Alogheli et al*.* used a 15 kcal mol^−1^ energy window and found that the conformer closest to the energy minimized X-ray structure averaged around 5 kcal mol^−1^ from the global minimum with the largest difference being 10.8 kcal mol^−1^ (almost the same data set as in this study and energies were calculated with OPLS-2005) [40].

Calculating the conformational energy penalty upon binding has been discussed rigorously in the literature and has recently been summarized by Peach [82]. Thus, no attempts tackling this subject will be made herein. However, as many modeling methods utilize an energy window for generating conformers, the width of this window is of great importance. Therefore, two energy differences were calculated and analyzed. The first between the global energy minimum and the energy minimized X-ray_ppw_ structure and the second between the global energy minimum and the MCMM-Exhaustive generated conformer closest to the X-ray_ppw_ structure. As mentioned in section "Generating a Conformation Similar to the X-ray_ppw_ Conformation", the conformer derived by energy minimizing the X-ray_ppw_ conformation should correspond to the minimum closest to the X-ray_ppw_ conformation. Therefore, with the aim of generating conformers closest to the bioactive conformation, the energy difference to this conformation could serve as an upper cut-off value for keeping conformers. The energy difference between the minimized X-ray_ppw_ conformation and the global minimum varied between 0 and 13.7 kcal mol^−1^, with a median value of 5.6 kcal mol^−1^ (Table S17). Therefore, the energy window of 15 kcal mol^−1^ used herein seems appropriate. It should be noted that only five out of the 45 macrocycles had energy differences exceeding 10 kcal mol^−1^.

As previously mentioned, the MCMM-Exhaustive searches were able to generate conformations similar to the X-ray_ppw_ structure (< 1 Å) for all but one of the 45 protein-macrocycle complexes, see Tables 7 and S13. When the energy difference between these conformations and the corresponding global energy minimum (generated by any method) was analyzed it varied between 0 and 21.4 kcal mol^−1^ with a median of 6.6 kcal mol^−1^ (see Table S17). The conformation closest to the X-ray_ppw_ conformation was found within 5 and 10 kcal mol^−1^ for 19 and 33 of the macrocycles, respectively.

Considering both energy differences, a 15 kcal mol^−1^ energy window for keeping conformers seems appropriate. However, there are many cases where an energy window of 10 kcal mol^−1^ or even 5 kcal mol^−1^, could be used instead.

## Conclusions

The present work has addressed macrocycle conformational sampling. We evaluated the performance of two of the commonly used, general-purpose conformational sampling methods and compared them with two more recent and specialized macrocycle sampling approaches. We also determined that using TNCG as the energy minimization method and combining it with wider ring closure distance settings (0–100 Å) and avoiding ring open bonds adjacent to chiral carbons for MCMM and MTLMOD may be used to enhance macrocycle sampling. Moreover, we have shown that generating a starting conformation from a SMILES-string to a 3D-structure, in some cases, might produce a conformation similar to the X-ray_ppw_ conformation. Thus, in all studies attempting to reproduce experimental data such as X-ray structures, the structural similarities between the starting conformation and the X-ray conformation should be analysed. Furthermore, as verified in the current study and by others, the stochastic nature of conformational sampling may influence the results. Consequently, we recommend assessing how stochastic elements inherent to a given search method affects these outcomes by either employing different starting conformations or different seeding.

Our comparative analysis of different sampling methods showed that, in most cases, the general conformational search methods (MCMM, MTLMOD) with standard and enhanced settings compared well with the more specialized macrocycle sampling methods (MD/LLMOD and PRIME-MCS). However, if the aim is to generate a large pool of conformers or a conformer close to the X-ray_ppw_ structure, any of the general methods could be recommended. The encouraging results of MCMM-Enhanced and MTLMOD-Enhanced suggest that conformational sampling of macrocycles might be manageable when it comes to the generation of conformers close to the X-ray_ppw_ conformation. However, if the aim is to identify the global minimum, more than 10,000 steps are required. Of the methods evaluated, the MD/LLMOD method performed the best in generating the global energy minimum.

## Electronic supplementary material

Below is the link to the electronic supplementary material.

Supplementary file1 (PDF 1812 kb)
