# Supplementary material for: Conformational analysis of macrocycles: comparing general and specialized methods
Source: J Comput Aided Mol Des. 2020 Jan 21;34(3):231–52. doi: 10.1007/s10822-020-00277-2 (PMC7036058; doi:10.1007/s10822-020-00277-2)
Supplement: Supplementary file 1 — Supplementary file1 (PDF 1812 kb) [file 10822_2020_277_MOESM1_ESM.pdf]

Supporting information

Conformational Analysis of Macrocycles: Comparing General and Specialized Methods

*Gustav Olanders, Hiba Alogheli, Peter Brandt†, Anders Karlén**

Department of Medicinal Chemistry, Uppsala University, BMC, Box 574, SE-751 23 Uppsala, Sweden

† Present address. Medicinal Chemistry, Research and Early Development Cardiovascular, Renal and Metabolism, BioPharmaceuticals R&D, AstraZeneca, Gothenburg, Sweden

*Corresponding author. Tel.: +46 18 471 42 93

E-mail address: anders.karlen@ilk.uu.se

CONTENTS

PRIME-MCS sampling-syntax.

Random seeding for MCMM and MTLMOD

Calculating the Number of Generated Conformers and Ring Conformations.

Method Optimization Using a Diverse Subset of 10 Macrocycles.

Selection of a Diverse Subset.

Conformational coverage and conformational energy distribution.

Figure S1. Principal Component Analysis (PCA) score-plot (full data set).

Figure S2. Principal Component Analysis (PCA) loading-plot. (full data set).

Figure S3. Dendrogram depicting of the initial nine clusters used for subset selection.

Figure S4. Energy distributions of all conformers for the 10 reference macrocycles in the MCMM conformational searches.

Table S1. Comparison of strategies for generating a starting conformation.

Table S2. Characteristics of the full data set.

Table S3. A Summary of Conformational Analysis Settings and Results for the Subset of 10 Diverse Macrocycles.

Table S4. Number of generated conformers (subset).

Table S5. Number of generated ring conformations (subset).

Table S6. Elapsed computational time in minutes (subset).

Table S7. Lowest energy conformer generated (kJ mol^{-1}, subset).

Table S8. Conformer with the lowest Root Mean Square Deviation (RMSD) in Ångtröm to the X-ray_{ppw} conformation (subset).

Table S9. Number of generated conformers (full data set).

Table S10. Number of generated ring conformations (full data set).

Table S11. Lowest energy conformer generated (kJ mol^{-1}, full data set).

Table S12. Elapsed computational time in minutes (full data set).

Table S13. Conformer with the lowest Root Mean Square Deviation (RMSD) in Ångtröm to the X-ray_{ppw} conformation (full data set).

Table S14. Mean values of the lowest heavy atom Root Mean Square Deviation (RMSD) in Ångtröm to the X-ray_{ppw} conformation (full data set).

Table S15. Conformer with the lowest Root Mean Square Deviation ($RMSD_{RING}$) in Ångtröm to the X-ray_{ppw} ring conformation (full data set).

Table S16. Mean values of the conformer with the lowest Root Mean Square Deviation ($RMSD_{RING}$) in Ångtröm to the X-ray_{ppw} ring conformation (full data set).

Table S17. Energy difference between: the energy minimized X-ray_{ppw} conformer to the global energy minimum and between the conformer closest to the X-ray_{ppw} conformation and the global energy minimum (full data set).

References

PRIME-MCS sampling-syntax.

PRIME-MCS was run from the command line in the extended sampling mode "thorough" (default from command line and corresponds to keyword --spinroot 10), with sampling of peptide bonds (-sample_peptide). Spinroot 1 and 10 corresponds to the sampling intensity "fast" and "thorough", generating up to 100 and 1 000 conformations, respectively. Random seeding was activated by adding the command "–specify USE_RANDOM_SEED=yes". The PRIME-MCS syntax is as follows:

$SCHRÖDINGER/run -FROM psp macro_sample.py "input_mae_file -sample_peptide -specify USE_RANDOM_SEED=yes

Random seeding for MCMM and MTLMOD

The SEED function was added before the MMOD function in the conformational search command file (.com-file). The SEED argument one was set to -1 and, the remaining arguments were set to zero.

Calculating the Number of Generated Conformers and Ring Conformations.

The number of generated conformers were extracted from the conformational search log files (.log files). The number of ring conformers generated by each method was investigated via the Redundant Conformation Elimination method implemented MacroModel. The heavy atoms in the macrocyclic ring were superimposed and redundant conformers were eliminated based on a maximum atom deviation cutoff of 0.5 Å. The energy window for keeping conformers was set to 62.8 kJ mol^{-1} (15.01 kcal mol^{-1}). MacroModel uses both geometry and energy to assess whether two conformations are identical or not. A pair of conformations differing more than 1 kJ mol^{-1} are considered as two unique conformations. However, conformers with identical ring conformations may have large conformational differences in the ring substituents giving rise to significant energy differences exceeding 1 kJ mol^{-1}. Consequently, MacroModel would incorrectly save both conformers as two unique ring conformations. Therefore, to only compare ring geometries, the energy comparison function was removed by changing the CRMS argument five from "0" to "-1" in the redundant conformational elimination command file (.com-file). Secondly, by default, the redundant conformation elimination terminates after storing 10 000 conformations. Since some of the conformational ensembles contained more than 10 000 ring conformations, the MULT function was added after the COMP function in the redundant conformational elimination command file (.com-file). The MULT argument one was set to 1 000 000 and, the remaining arguments were set to zero.

Method Optimization Using a Diverse Subset of 10 Macrocycles.

For conformational sampling of macrocyclic rings using MCMM and MTLMOD, the argument defining where the ring is opened (and then re-closed) is central. Both avoiding having the ring opening bond adjacent to stereocenters and widening the ring closure criterion could affect the search performance. The default ring closure criterion is 0.5 to 2.5 Å, however, it is recommended to widen the ring closure criterion for large rings to 0.1 to 5.0 Å.[1] As seen in Table S3 and Table S5, increasing

the ring closure from 0.5 – 2.5 Å to 0.1 – 5 Å for MCMM increased the number of ring conformations generated as well as the number of conformers close to the X-ray_{ppw} conformation (from 7 to 9, below 2 Å, see Table S3). Increasing the ring closure from default 0.5 – 2.5 Å to 0 – 100 Å, increased the number of generated ring conformations from 2 482 to 9 326, over the 10 macrocycles, as well as the ability to generate conformers close to the X-ray_{ppw} conformation (< 2 Å, see Table S3 and Table S8). When using the automatic set-up it resulted in at least one of the ring opening atoms being a stereocenter for 7 of the 10 macrocycles. Manually re-defining this bond ("MCMM moved" in Table S3) increased the number of identified conformers and ring conformations slightly as compared to when using automatic set-up. Oppositely, the total number of identified conformers decreased when the ring closure criterion was widened (0 – 100 Å). Finally, for both MCMM and MTLMOD we evaluated the performance when changing both the ring opening width and placement. When these settings were compared to standard settings the total number of conformers identified decrease slightly for MCMM but increased for MTLMOD. The aim of this part of the study was to investigate if we could enhance standard settings in MCMM and MTLMOD searches to improve macrocyclic sampling. The combination of increasing the ring opening width and re-defining the ring opening bond provided the highest number of ring conformations and also at least one conformer within 2 Å RMSD to the X-ray_{ppw} conformation for all 10 macrocycles. We reasoned that the ability to generate many ring conformations in a conformation analysis of macrocycles is of key importance and therefore these settings were used in all subsequent studies and are called MCMM enhanced and MTLMOD enhanced**.**

Selection of a Diverse Subset.

To investigate how different parameters affect the conformational search, ten diverse macrocycles were chosen from the 47 macrocycles in the Alogheli *et al.* data set as a subset to represent the full data. A principal component analysis (PCA), based on eight descriptors, was performed. The number of principal components used for selecting macrocycles was set to three, explaining 84% of the variance in the data set (see Figure S1 for the score plot and Figure S2 for the loading plot). The descriptors used for the PCA corresponds to size and flexibility (molecular weight, ring size, number of torsion angles sampled), and polarity/lipophilicity (polar surface area, the number of hydrogen bond acceptors and donors, and a $logP_{o/w}$). These descriptors were calculated using QikProp[2] except for permanently charged molecules where Instant JChem[3] were used. In addition, the lowest obtained RMSD value to the X-ray_{ppw} ligand from the MCMM conformational search used for choosing starting conformation, and the number of generated conformations from the same search was included to describe the difficulty to generate a conformer close to the X-ray_{ppw} conformation, and the flexibility of the macrocycles. Nine clusters were generated by hierarchical clustering and one representative macrocycle from each cluster was picked, see Figure S3. Thereafter, one additional macrocycle (3I6O) from the center of the PCA was added. In cluster 6, the macrocycle with the PDB code 1TPS was identified as an outlier and was therefore not considered for inclusion; instead, 2J9M was added, see Figure S2.

Conformational coverage and conformational energy distribution.

As shown earlier (in section "*3.4.1. Total Number of Conformers Generated*" in the paper), many more conformers can be generated when increasing the number of search steps from 10 000 to 1 000 000 for most of the macrocycles, but does this also apply to the number of ring conformations? Hypothetically, the macrocyclization could constrain the torsion angles in the macrocyclic ring to such degree that only a small number of possible ring conformations remains. Given this scenario, a short 10 000 step conformational search might be able to generate all possible ring conformations within the given energy window. However, MCMM, MTLMOD, MMBS, PRIME-MCS, MCMM-enhanced and, MTLMOD-enhanced found 2 – 5% of the total number of ring conformations generated by MCMM exhaustive, see Table S10. Thus, the number of ring conformations follows the same pattern as the number of generated conformers where many more ring conformations are identified by a more rigorous sampling.

Comparing the energy distribution for the longer and shorter search in Figure S1, a higher percent of the conformational ensemble contains high-energy conformers comparing the longer and shorter search. Thus, a longer search mostly generates high-energy conformations. Hypothetically, there are more high-energy minima than low energy minima to find on the potential energy surface. Thus, the more exhaustive search is, therefore, more likely to be a better representation of the potential energy surface. Looking further at the energy distribution for all conformers of the 10 macrocycles (sorted by energy) the vast majority of the conformers had a relative energy above 10 kcal mol^{-1} (see Figure S4). For the MCMM enhanced search (Figure S4A) ca. 50% of the conformers had an energy below 10 kcal mol^{-1} while for the MCMM exhaustive search (Figure S4B) ca. 30% of the conformers had an energy below 10 kcal mol^{-1}. Thus, the majority of the conformers in both searches are of high energy (>10 kcal mol^{-1}). It can also be noted that the curvature of slopes in Figure S4A and Figure S4B are not identical. The shorter search generates relatively more low-energy conformations. One explanation for this could be that the MCMM method is very efficient in generating low energy conformers early in the search. This may originate from the MCMM search algorithm, where low-energy conformers are more frequently chosen as starting conformers during the search.[4]

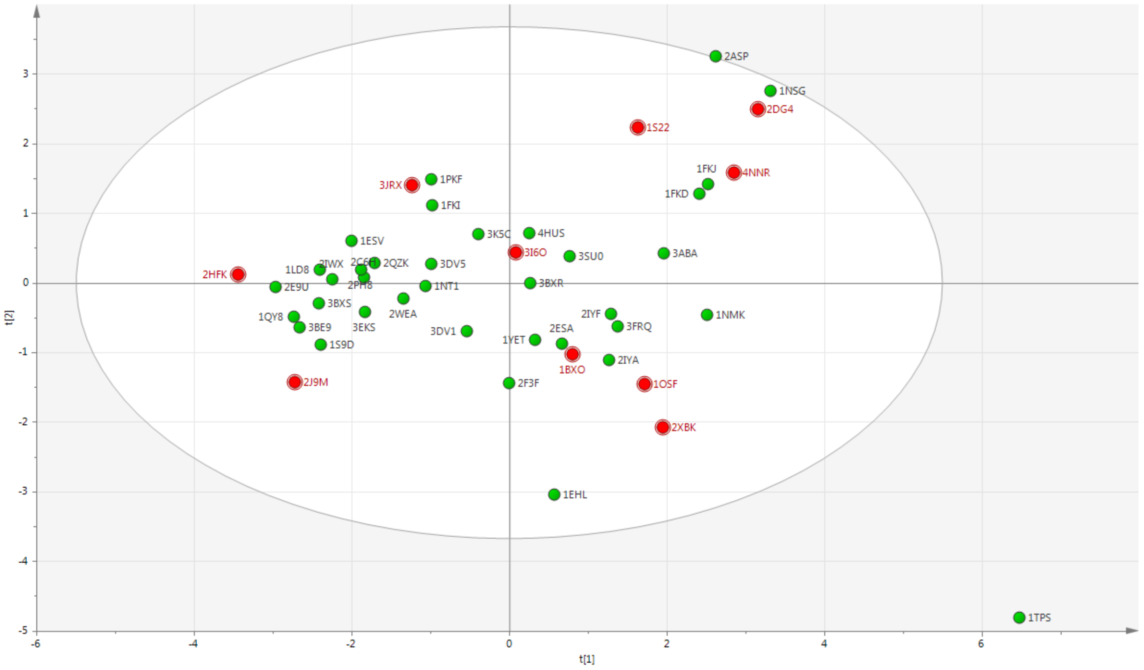

Figure S1. Principal Component Analysis (PCA) score-plot (full data set). The red marked macrocycles were selected for initial parameter testing. The ellipse corresponds to Hotelling´s T2 distribution (95%).

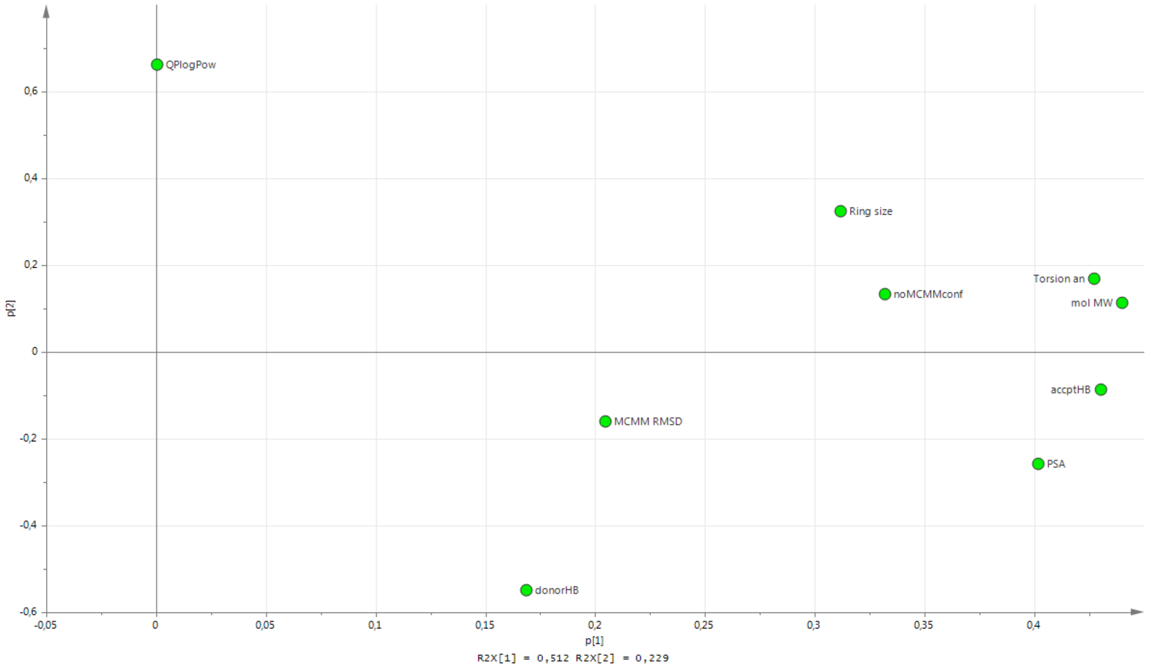

Figure S2. Principal Component Analysis (PCA) loading-plot. (full data set).

Torsion an: Number of torsion angles sampled during the MCMM and MTLMOD conformational searches. donorHB: Number of hydrogen bond donors. accptHB: Number of hydrogen bond acceptors. QPlogPow: Calculated octanol/water partition coefficient. PSA: Polar surface area. MCMM RMSD: the lowest obtained RMSD value to the X-ray$_{ppw}$ ligand from the MCMM conformational search used for choosing starting conformation. mol MW: molecular weight. noMCMMconf: Number of conformations generated during the MCMM conformational search used for choosing starting conformation. Ring size: number of macrocyclic ring atoms (calculated by hand).

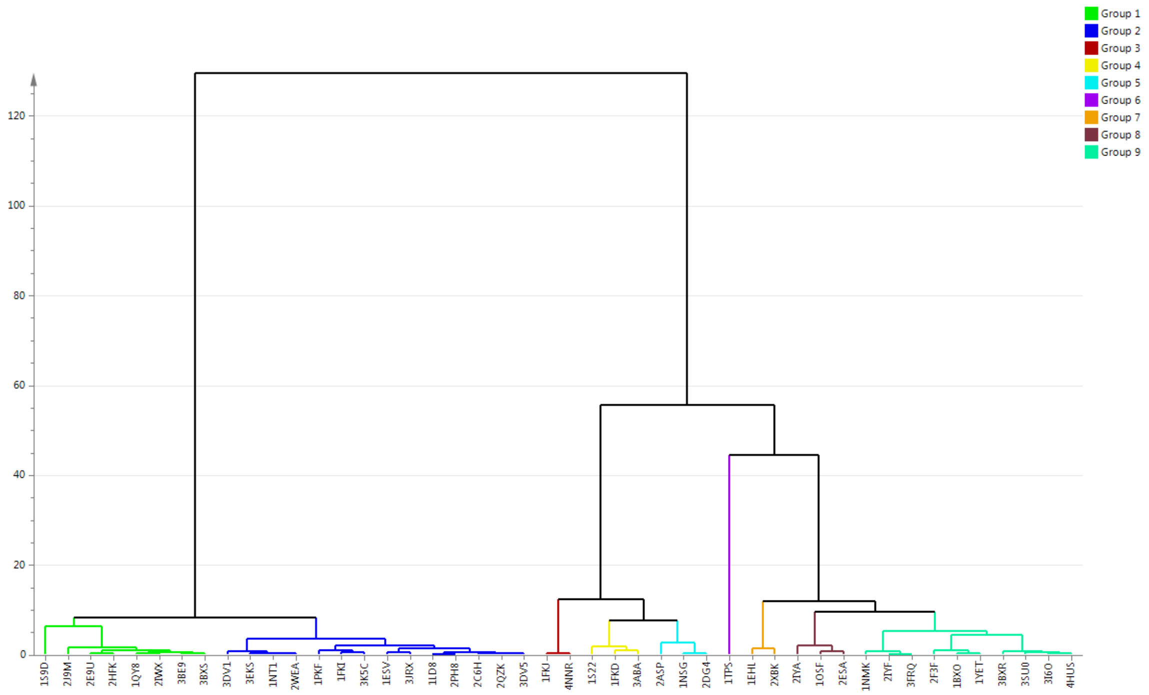

Figure S3. Dendrogram depicting of the initial nine clusters used for subset selection. One macrocycle was picked from each cluster.

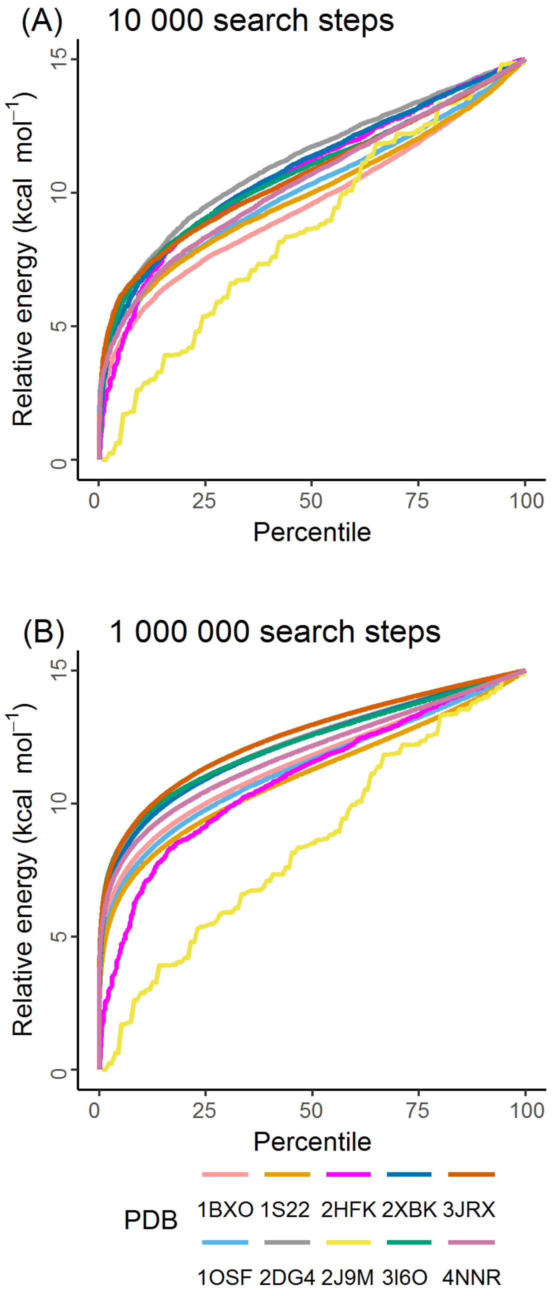

Figure S4. Energy distributions of all conformers for the 10 reference macrocycles in the MCMM conformational searches using (A) 10 000 (MCMM enhanced) and (B) 1 million search steps (MCMM exhaustive) showing that low energy conformations are more easily found than high energy conformations using MCMM (see the increased slope of initial curves).

Table S1. Comparison of strategies for generating a starting conformation.

	All heavy atoms RMSD (Å)			$RMSD_{RING}$ (Å)			Torsional RMSD (Deg)				Number of dihedrals differing more than:					
											120°			60°		
PDB	Energy mini. X-ray	Starting conf.	SMILE conf.	Energy mini. X-ray	Starting conf.	SMILE conf.	Energy mini. X-ray	Starting conf.	SMILE Conf.	#Dihedrals (Varied torsions)	Energy mini. X-ray	Starting conf.	SMILE Conf.	Energy mini. X-ray	Starting Conf	SMILE Conf.
1BXO	1.34	4.71	3.10	0.28	0.64	1.2	16.8	68.1	64.3	53 (24)	0	6	6	1	16	15
1EHL	0.17	2.3	1.82	0.12	0.95	0.8	4.7	68.8	61.8	75 (24)	0	5	4	0	28	19
1ESV	0.23	2.75	1.20	0.1	1.22	0.46	6.9	80.0	85.8	47 (21)	0	3	7	0	20	15
1FKD	0.29	3.72	2.66	0.1	0.44	1.34	5.3	44.3	54.5	103 (41)	0	5	6	0	10	18
1FKI	0.25	3.39	2.69	0.16	2.26	1.74	12.9	90.7	75.8	37 (24)	0	6	3	0	21	15
1LD8	0.71	2.79	2.66	0.38	1.12	1.04	15.4	65.7	61.1	72 (15)	0	5	5	0	11	7
1NMK	0.4	4.49	4.35	0.09	1.66	1.37	9.2	67.0	74.7	78 (34)	0	6	11	0	13	21
1NSG	0.32	3.69	2.71	0.25	1.06	1.45	5.6	43.5	78.5	107 (46)	0	2	10	0	14	40
1NT1	0.57	4.29	2.87	0.36	1.66	1.27	8.7	74.7	48.6	54 (24)	0	3	2	0	15	5
1OSF	0.67	4.43	3.20	0.09	1.58	1.56	7.0	73.4	82.8	65 (23)	0	11	11	0	16	27
1PKF	0.35	3.68	1.82	0.15	1.41	0.92	8.4	64.3	61.7	53 (21)	0	2	3	0	13	13
1QY8	0.35	3.18	1.86	0.2	1.51	0.69	12.2	68.9	55.2	42 (11)	0	3	4	0	8	4
1S22	0.63	4.8	4.75	0.25	1.71	1.29	7.3	67.1	77.3	84 (38)	0	4	6	0	21	25
1S9D	0.21	1.77	**0.67**	0.08	0.95	0.42	5.6	92.3	55.1	31 (15)	0	5	2	0	10	6
1TPS	1.36	5.73	4.81	0.27	1.4	0.86	10.0	89.9	84.4	95 (47)	0	14	12	0	36	34
2ASP	0.37	5.94	3.44	0.22	1.92	2.01	5.2	64.9	69.8	98 (40)	0	10	11	0	26	29
2C6H	1.12	3.22	1.62	0.12	0.74	0.6	17.2	87.0	55.3	53 (20)	0	6	2	0	25	11
2DG4	0.53	4.94	2.77	0.37	1.51	1.7	5.9	39.8	79.1	106 (45)	0	1	8	0	17	41
2E9U	0.93	2.24	**0.95**	0.52	0.95	0.83	31.6	60.0	56.4	37 (11)	0	2	5	3	8	6
2ESA	0.22	3.76	3.13	0.12	1.49	1.57	3.4	74.3	70.2	61 (20)	0	5	7	0	16	17
2F3F	0.93	4.63	2.50	0.15	0.72	0.88	13.5	70.0	79.8	45 (26)	0	1	6	1	17	13
2HFK	0.13	1.95	**0.52**	0.08	0.82	0.08	5.2	105.9	31.3	29 (12)	0	6	0	0	18	2
2IWX	0.2	2.89	1.36	0.15	1.53	0.97	4.4	94.8	73.2	32 (14)	0	2	2	0	10	4
2IYA	0.37	4.79	2.75	0.13	0.79	0.85	5.6	70.7	59.4	96 (32)	0	9	6	0	32	22
2J9M	0.13	1.42	1.08	0.06	0.59	0.52	5.3	53.1	58.3	33 (8)	0	2	2	0	4	5
2PH8	0.6	3.17	2.18	0.34	1.08	0.77	10.1	74.3	80.0	50 (13)	0	6	8	0	17	12
2QZK	0.79	3.67	3.30	0.21	1.33	1.22	15.0	65.5	73.7	71 (13)	0	8	9	0	13	15
2WEA	0.39	4.43	2.46	0.08	0.69	0.1	7.0	64.6	54.9	62 (16)	0	4	4	0	15	8
2XBK	0.68	3.3	1.33	0.32	1.23	0.81	15.4	45.8	37.6	73 (33)	0	3	2	0	8	7
2XYT	0.23	3.15	1.50	0.14	1.49	0.69	5.9	48.7	35.3	108 (19)	0	3	1	0	16	10
3ABA	0.43	4.12	2.08	0.24	1.31	1.28	10.0	57.2	26.0	56 (34)	0	2	0	0	12	3
3BE9	0.4	1.63	**0.64**	0.15	0.76	0.6	10.1	43.1	35.3	57 (12)	0	2	2	0	4	5
3BXR 15	0.58	4.87	3.63	0.08	0.65	0.54	8.9	87.8	77.3	72 (29)	0	8	6	0	23	23
3BXR 16				0.18	0.69	0.87										
3BXS A	0.38	1.97	1.30	0.23	1.26	0.98	14.8	69.9	77.3	33 (14)	0	3	3	0	8	6
3BXS B	0.38	1.81	1.57	0.13	0.82	0.87	13.2	76.1	85.3	33 (14)	0	6	7	0	12	14
3DV1	0.98	4.18	2.61	0.12	0.89	0.9	16.1	79.8	68.1	35 (23)	0	2	4	1	18	9
3DV5	0.72	4.45	1.95	0.17	0.78	0.66	11.1	71.9	73.9	49 (22)	0	1	5	0	19	10
3EKS	0.26	1.97	1.46	0.03	0.38	0.05	5.0	71.0	32.5	76 (21)	0	7	1	0	16	3
3FRQ	0.49	3.22	2.92	0.16	0.84	0.96	5.7	69.0	50.0	100 (35)	0	7	3	0	34	22
3I6O	1.03	5.1	2.43	0.13	0.48	0.77	9.8	60.7	62.7	76 (28)	0	4	5	0	10	21
3JRX	0.23	3.11	1.54	0.14	1.24	0.65	5.5	78.0	45.1	72 (23)	0	12	0	0	27	16
3K5C	0.76	4.39	3.26	0.19	0.58	1.19	11.7	67.7	71.4	80 (20)	0	9	7	0	17	17
3SU0	1.12	4.18	2.30	0.13	0.87	0.85	15.5	59.2	54.7	86 (29)	0	4	3	2	18	17
4HUS	0.64	3.25	1.57	0.41	1.89	0.91	10.3	78.4	64.3	56 (26)	0	9	5	0	21	11
4NNR	0.55	4.76	3.99	0.28	1.71	1.22	8.9	57.6	64.9	100 (41)	0	6	10	0	20	20
< 1 Å[a]	40	0	4	46	22	30	NA[d]	NA[d]	NA[d]	NA[d]	NA[d]	NA[d]	NA[d]	NA[d]	NA[d]	NA[d]
1 – 2 Å[b]	5	7	15	0	23	15	NA[d]	NA[d]	NA[d]	NA[d]	NA[d]	NA[d]	NA[d]	NA[d]	NA[d]	NA[d]
> 2 Å[c]	0	38	26	0	1	1	NA[d]	NA[d]	NA[d]	NA[d]	NA[d]	NA[d]	NA[d]	NA[d]	NA[d]	NA[d]
% < 2 Å[e]	0	15.6	42.2	100	97.8	97.8	NA[d]	NA[d]	NA[d]	NA[d]	NA[d]	NA[d]	NA[d]	NA[d]	NA[d]	NA[d]
median	0.43	3.68	2.4	0.15	1.07	0.88	8.9	68.9	64.3	62 (23)	0	5	5	0.2	16	14
average	0.54	3.61	2.3	0.19	1.12	0.94	9.9	69.0	62.8	65.1 (24.5)	0	5.1	5.0	0	16.7	14.7

[a]Number of cases where the method generated a conformer with an RMSD value less than 1 Å to the X-ray conformer. [b]Number of cases where the method generated a conformer with an RMSD value between 1 and 2 Å to the X-ray conformer. [c]Number of cases where the method generated a conformer with an RMSD value greater than 2 Å to the X-ray conformer. [d]Not Applicable. [e]Percent below 2 Å RMSD.

Table S2. Characteristics of the full data set.

PDB	PDB resolution (Å)	Ring size[a]	#Torsion angles sampled[b]	Molecular weight[c]	donorHB[d]	accptHB[e]	$clogP_{o/w}$[f]	PSA[g]
1BXO	0.95	15	24	639	3.5	15.5	0.63	193
1EHL	2.4	14	24	546	5.0	19.6	-1.77	236
1ESV	2	16	21	422	2.0	6.0	4.16	99
1FKD	1.72	21	41	808	2.0	18.7	3.73	194
1FKI	2.2	21	24	438	0.0	9.0	3.27	101
1LD8	1.8	16	15	435	1.0	8.0	2.79	85
1NMK	2.1	22	34	741	4.5	15.9	2.82	229
1NSG	2.2	29	46	928	1.0	19.0	6.84	208
1NT1	2	17	24	505	1.3	9.5	0.93	127
1OSF	1.75	19	23	617	4.3	16.4	1.57	200
1PKF	2.1	16	21	492	0.0	6.9	5.16	106
1QY8	1.85	14	11	365	2.0	7.5	1.42	108
1S22	1.6	25	38	696	0.0	16.1	4.05	167
1S9D	1.8	13	15	280	2.0	5.4	1.82	80
1TPS	1.9	19	47	1 041	9.3	26.9	-2.59	411
2ASP	1.64	26	40	889	0.0	18.9	6.51	138
2C6H	2.35	12	20	454	1.0	11.1	2.61	80
2DG4	1.7	29	45	914	1.0	19.0	6.27	209
2E9U	2	15	11	349	2.0	5.3	2.59	84
2ESA	1.9	19	20	561	3.3	14.1	1.71	182
2F3F	2.3	15	26	515	5.0	11.7	0.51	124
2HFK	1.79	12	12	296	1.0	5.7	2.83	71
2IWX	1.5	15	14	353	2.0	5.5	2.32	94
2IYA	1.7	14	32	688	2.0	20.6	1.79	138
2J9M	2.5	13	8	384	3.0	7.5	1.14	97
2PH8	1.7	16	13	460	2.0	8.5	3.02	87
2QZK	1.8	16	13	530	3.0	7.0	4.96	116
2WEA	1.25	12	16	553	2.3	11.3	2.98	134
2XBK	1.95	24	33	650	7.0	15.7	-0.32	220
2XYT	2.05	18	19	611	2.0	9.0	2	81
3ABA	1.8	28	34	623	6.0	12.9	3.27	169
3BE9	2	14	12	363	3.0	6.5	2.63	97
3BXR	1.6	16	29	678	2.8	11.8	2.05	155
3BXS	1.6	16	14	362	1.5	6.3	1.60	122
3DV1	2.1	15	23	440	4.0	9.2	1.45	118
3DV5	2.1	16	22	488	3.0	8.7	3.18	75
3EKS	1.8	11	21	508	3.0	9.0	3.36	123
3FRQ	1.76	14	35	734	4.0	19.2	3.05	170
3I6O	1.17	13	28	631	2.0	13.6	3.84	123
3JRX	2.5	16	23	521	1.0	9.3	4.36	84
3K5C	2.12	16	20	572	4.0	8.7	5.13	87
3SU0	1.16	15	29	732	1.5	14.0	3.32	201
4HUS	2.36	23	26	526	1.0	12.2	2.26	156
4NNR	1.98	23	41	804	1.0	17.0	5.58	184
Average	1.88	17.5	24.7	571	2.5	12.0	2.75	142
Median	1.88	16	23	538	2.0	11.2	2.80	124
Max	2.50	29	47	1041	9.3	26.9	6.84	411
Min	0.95	11	8	280	0.0	5.3	-2.59	71

[a]Macrocyclic ring size. [b]Number of torsion angles sampled during the conformational search. [c]Molecular weight. [d]Number of hydrogen bond donors. [e]Number of hydrogen bond acceptors. [f]Calculated logP octanol/water. [g]Polar surface area.

Table S3. A summary of conformational analysis settings and results for the subset of 10 diverse macrocycles.

method	ring opening	ring closure distance (Å)	no. conf[a]	no.unique ring conf[b]	user time (min)[c]	global energy minimum found for no. macrocycles[d]	best fit conformation RMSD (Å)[e]		
							< 1 Å	1 Å – 2 Å	> 2 Å
MCMM	standard	0.5 – 2.5	40 507	2 482	666	4	7	0	3
MCMM	standard	0.1 – 5.0	40 946	5 158	646	3	8	1	1
MCMM	standard	0 – 100	36 034	9 326	813	5	7	3	0
MCMM	moved	0.5 – 2.5	41 538	2 811	614	3	6	2	2
MCMM enhanced	moved	0 – 100	38 037	9 402	800	5	7	3	0
MTLMOD	standard	0.5 – 2.5	29 417	4 082	714	4	6	1	3
MTLMOD enhanced	moved	0 – 100	32 489	7 574	704	3	8	2	0

[a]The sum total of conformers generated for the 10 macrocycles. [b]The sum total of unique ring conformations generated for 10 macrocycles. [c]The sum total of computational time (minutes) consumption for conformational analysis of 10 macrocycles. [d]Number of macrocycles where the lowest energy conformer was identified or a conformer with an energy difference no greater than 1 kJ mol^{-1}. [e]RMSD for the conformer identified with the lowest RMSD value to the X-ray ligand after protein preparation treatment. The conformers are, dependent on their RMSD values, divided into three different groups with RMSD values: below 1 Å, between 1 Å - 2 Å, and greater than 2 Å.

Table S4. Number of generated conformers (subset).

PDB	MCMM[a]	MCMM ring closure 0.1 – 5.0 Å	MCMM ring closure 0 – 100 Å	MCMM no ring closure adjacent to stereocenter[a]	MCMM enhanced	MTLMOD	MTLMOD enhanced
1BXO	6 074	5 982	5 714	6 259	5 944	3 794	4 444
1OSF	6 053	5 998	5 559	6 015	5 561	3 747	4 517
1S22	6 258	6 507	5 850	6 258	5 850	3 978	4 801
2DG4	3 882	4 095	2 610	3 882	2 610	3 767	2 940
2HFK	733	740	733	714	716	724	724
2J9M	121	122	123	121	123	117	119
2XBK	3 239	3 318	2 468	3 239	2 468	3 632	3 372
3I6O	4 906	5 025	5 448	4 906	5 448	3 321	3 906
3JRX	3 713	3 860	3 984	4 425	4 823	3 104	4 043
4NNR	5 528	5 299	3 545	5 719	4 494	3 233	3 623
sum	40 507	40 946	36 034	41 538	38 037	29 417	32 489

[a]Standard ring closure of 0.5 -2.5

Table S5. Number of generated ring conformations (subset).

PDB	MCMM[a]	MCMM ring closure 0.1 – 5.0 Å	MCMM ring closure 0 – 100 Å	MCMM no ring closure adjacent to stereocenters[a]	MCMM enhanced	MTLMOD	MTLMOD enhanced
1BXO	129	283	456	162	513	141	456
1OSF	206	550	1 019	236	982	191	796
1S22	576	1 306	2 747	576	2 747	714	1 901
2DG4	309	684	1 186	309	1 186	1 444	942
2HFK	108	112	108	104	111	109	105
2J9M	54	54	56	54	56	54	54
2XBK	397	610	991	397	991	681	1 203
3I6O	69	171	320	69	320	79	280
3JRX	444	848	1 254	689	1 280	543	1 027
4NNR	190	540	1 189	215	1 216	126	810
sum	2 482	5 158	9 326	2 811	9 402	4 082	7 574

[a]Standard ring closure of 0.5 -2.5

Table S6. Elapsed computational time in minutes (subset).

PDB	MCMM[a]	MCMM ring closure 0.1 – 5.0 Å	MCMM ring closure 0 – 100 Å	MCMM no ring closure adjacent to stereocenter[a]	MCMM enhanced	MTLMOD	MTLMOD enhanced
1BXO	111	93	99	83	117	105	94
1OSF	71	49	65	70	71	67	56
1S22	52	57	81	52	81	63	64
2DG4	130	125	179	130	179	163	167
2HFK	10	9	8	8	11	12	11
2J9M	10	7	9	10	9	9	8
2XBK	47	42	65	47	65	51	52
3I6O	115	102	113	115	113	117	106
3JRX	29	31	48	27	39	33	34
4NNR	92	130	147	73	116	92	111
sum	666	646	813	614	800	714	704

[a]Standard ring closure of 0.5 -2.5

Table S7. Lowest energy conformer generated (kJ mol^{-1}, subset).

PDB	MCMM[a]	MCMM ring closure 0.1 – 5.0 Å	MCMM ring closure 0 – 100 Å	MCMM no ring closure adjacent to stereocenter[a]	MCMM enhanced	MTLMOD	MTLMOD enhanced
1BXO	-760.4	-759.9	-763.2	-760.9	-760.5	-760.8	-749.2
1OSF	-31.4	-35.0	-34.0	-35.4	-37.8	-35.7	-34.4
1S22	-434.5	-439.8	-436.9	-434.5	-436.9	-432.9	-438.7
2DG4	82.6	87.9	92.0	82.6	92.0	109.2	86.4
2HFK	-84.5	-84.6	-84.5	-84.5	-84.5	-84.5	-84.6
2J9M	-847.0	-847.0	-847.0	-847.0	-847.0	-847.0	-847.0
2XBK	-1 189.2	-1 188.1	-1 189.2	-1 189.2	-1 189.2	-1 189.2	-1 189.2
3I6O	-333.7	-335.0	-333.9	-333.7	-333.9	-336.8	-336.9
3JRX	-24.3	-24.3	-24.3	-22.3	-24.3	-24.3	-22.4
4NNR	122.7	120.8	118.1	124.7	117.2	115.3	107.0

[a]Standard ring closure of 0.5 -2.5.

Table S8. Conformer with the lowest Root Mean Square Deviation (RMSD) in Ångtröm to the X-ray conformation (subset).

PDB	energy minimized X-ray ligand	MCMM[a]	MCMM ring closure 0.1 – 5.0 Å	MCMM ring closure 0 – 100 Å	MCMM no ring closure adjacent to stereocenter[a]	MCMM enhanced	MTLMOD	MTLMOD enhanced
1BXO	1.34	0.63	0.88	0.79	1	0.89	0.84	0.78
1OSF	0.67	2.06	0.76	1.23	1.53	0.5	2.2	0.57
1S22	0.63	2.08	1.61	1.08	2.08	1.08	2.87	1.7
2DG4	0.53	0.47	0.47	1.23	0.47	1.23	1.61	0.59
2HFK	0.13	0.13	0.13	0.13	0.13	0.13	0.13	0.13
2J9M	0.13	0.78	0.78	0.78	0.78	0.78	0.78	0.78
2XBK	0.68	0.71	0.6	0.61	0.71	0.61	0.8	0.67
3I6O	1.03	0.81	0.81	0.75	0.81	0.75	0.8	0.81
3JRX	0.26	0.34	0.32	0.39	0.26	0.27	0.84	0.27
4NNR	0.55	2.28	2.31	0.92	2.31	1.68	2.6	1.38
< 1 Å	8	7	8	7	6	7	6	8
1 Å – 2 Å	2	0	1	3	2	3	1	2
> 2 Å	0	3	1	0	2	0	3	0

[a]Standard ring closure of 0.5 -2.5

Table S9. Number of generated conformers (full data set).

		Number of Generated Conformers						
	**METHOD NAME**	**MCMM**	**MCMM-ENHANCED**	**MCMM-EXHAUSTIVE**	**PRIME-MCS**	**MD/LLMOD**	**MTLMOD**	**MTLMOD-ENHANCED**
**PDB CODE**								
	**0**	6 043	6 141	359 789	897	1 233	3 898	4 261
**1BXO**	**1**	6 014	5 345		875		3 825	3 829
	**2**	5 912	5 963		891		3 781	4 495
	**0**	1 909	2 024	4 490	150	143	1 761	2 040
**1EHL**	**1**	2 078	2 017		158		1 903	1 960
	**2**	1 943	2 041		149		1 722	2 050
	**0**	1 640	1 874	3 417	311	259	1 703	1 870
**1ESV**	**1**	1 640	1 849		306		1 698	1 867
	**2**	1 632	1 867		310		1 759	1 843
	**0**	5 512	3 813	283 599	991	1 007	2 818	3 560
**1FKD**	**1**	5 744	2 397		993		2 970	3 400
	**2**	5 468	3 295		990		3 579	2 807
	**0**	3 433	6 163	480 490	997	3 349	5 669	6 202
**1FKI**	**1**	3 511	6 208		994		5 620	6 331
	**2**	3 449	6 274		995		5 715	6 269
	**0**	114	118	139	110	51	111	114
**1LD8**	**1**	114	116		113		119	116
	**2**	115	118		112		117	115
	**0**	6 557	3 816	400 310	991	2 638	3 560	3 620
**1NMK**	**1**	6 303	4 220		991		3 781	3 756
	**2**	6 400	3 423		993		3 752	3 581
	**0**	4 505	1 607	190 761	994	1 583	2 792	2 712
**1NSG**	**1**	4 564	2 658		992		3 228	3 525
	**2**	4 505	2 519		993		3 306	2 781
	**0**	2 704	3 548	18 545	751	848	2 602	3 168
**1NT1**	**1**	2 658	3 550		754		2 560	3 149
	**2**	2 537	3 657		752		2 336	3 166
	**0**	5 839	5 935	360 327	946	1 330	3 605	4 569
**1OSF**	**1**	6 283	5 816		943		3 742	4 486
	**2**	5 606	6 106		943		3 691	4 595
	**0**	5 325	6 068	210 833	974	1 237	3 602	4 875
**1PKF**	**1**	5 181	6 049		970		3 598	4 781
	**2**	5 271	5 820		981		3 580	4 565
	**0**	649	641	941	128	113	634	629
**1QY8**	**1**	645	640		130		636	647
	**2**	640	641		128		631	644
	**0**	6 304	5 853	498 213	990	2 662	3 935	4 763
**1S22**	**1**	6 290	5 928		983		4 053	4 710
	**2**	6 218	5 794		989		4 157	4 767
	**0**	1 737	1 720	2 199	169	75	1 748	1 777
**1S9D**	**1**	1 696	1 699		169		1 704	1 744
	**2**	1 714	1 726		172		1 724	1 765
	**0**	5 255	3 971	361 525	981	2 690	3 672	3 092
**1TPS**	**1**	5 526	4 260		979		3 188	3 302
	**2**	5 750	4 034		986		2 883	3 413
	**0**	5 481	3 427	243 815	999	1 807	4 036	3 383
**2ASP**	**1**	4 671	3 224		998		3 783	3 580
	**2**	4 687	3 090		999		3 645	3 841
	**0**	2 016	2 076	6 380	558	254	1 921	2 104
**2C6H**	**1**	2 267	2 128		559		1 917	2 032
	**2**	2 212	2 127		567		1 892	2 093
	**0**	4 359	3 132	180 720	990	1 199	3 211	3 183
**2DG4**	**1**	4 592	2 322		996		3 224	3 552
	**2**	3 953	2 479		986		3 200	3 471
	**0**	636	650	739	380	89	650	663
**2E9U**	**1**	638	648		381		651	666
	**2**	634	650		382		653	663
	**0**	4 422	4 603	88 513	932	828	2 541	3 633
**2ESA**	**1**	4 433	4 644		922		2 431	3 604
	**2**	4 562	4 541		932		2 529	3 550
	**0**	5 787	4 343	286 396	849	2 323	4 162	4 515
**2F3F**	**1**	5 809	4 595		846		4 183	4 366
	**2**	5 924	4 598		835		3 780	4 484
	**0**	738	720	836	272	134	715	717
**2HFK**	**1**	740	714		272		724	721
	**2**	743	702		278		715	717
	**0**	3 940	4 261	12 375	702	189	4 020	4 303
**2IWX**	**1**	3 895	4 237		712		3 955	4 305
	**2**	3 948	4 233		704		4 015	4 314
	**0**	2 764	3 327	111 055	942	894	3 088	3 084
**2IYA**	**1**	2 592	2 948		933		3 267	3 031
	**2**	2 278	2 970		935		2 896	3 220
	**0**	120	121	135	122	16	119	121
**2J9M**	**1**	123	122		125		116	121
	**2**	123	122		123		119	121
	**0**	2 791	3 084	6 002	522	671	2 222	2 751
**2PH8**	**1**	2 849	3 065		508		2 091	2 788
	**2**	2 819	3 084		523		2 137	2 723

	**0**	1 587	1 679	2 413	597	554	1 386	1 576
**2QZK**	**1**	1 592	1 632		587		1 318	1 628
	**2**	1 575	1 649		618		1 346	1 595
	**0**	2 736	2 612	6 167	453	609	1 903	2 231
**2WEA**	**1**	2 719	2 608		436		1 979	2 207
	**2**	2 770	2 561		449		2 015	2 221
	**0**	3 339	2 437	156 479	761	1 222	3 545	3 429
**2XBK**	**1**	3 268	2 471		765		3 547	3 402
	**2**	3 267	2 676		762		3 476	3 387
	**0**	1 382	1 157	2 715	148	106	891	1 295
**2XYT**	**1**	1 169	1 416		145		902	1 029
	**2**	1 345	1 167		154		1 013	1 040
	**0**	6 362	2 262	234 459	967	1 642	3 697	3 226
**3ABA**	**1**	6 248	2 427		976		3 744	3 203
	**2**	5 986	2 493		970		3 763	3 613
	**0**	357	368	384	299	39	354	370
**3BE9**	**1**	367	366		310		353	370
	**2**	363	370		301		347	371
	**0**	4 214	6 045	338 113	875	922	3 181	4 655
**3BXR**	**1**	4 054	6 013		867		3 197	4 622
	**2**	4 445	6 099		880		2 927	4 641
	**0**	848	873	1 122	600	144	801	855
**3BXS**	**1**	858	861		602		790	851
	**2**	844	867		600		792	863
	**0**	6 114	7 079	578 651	962	2 889	5 089	6 089
**3DV1**	**1**	6 019	6 800		967		5 189	6 175
	**2**	6 332	7 121		970		5 146	6 071
	**0**	5 006	6 240	362 879	989	1 584	3 640	4 556
**3DV5**	**1**	5 231	5 970		994		3 454	4 616
	**2**	4 584	5 944		992		3 559	4 577
	**0**	815	791	1 073	302	69	637	692
**3EKS**	**1**	822	795		296		557	714
	**2**	777	796		296		656	729
	**0**	3 904	4 189	191 100	965	816	3 482	3 261
**3FRQ**	**1**	4 009	4 106		963		3 336	3 456
	**2**	3 750	4 026		957		3 476	3 461
	**0**	5 062	5 461	305 225	857	911	3 384	3 872
**3I6O**	**1**	5 241	4 913		863		3 554	3 992
	**2**	5 524	5 089		858		3 040	4 027
	**0**	3 855	4 787	82 662	907	677	3 137	3 840
**3JRX**	**1**	4 353	4 738		913		3 151	4 101
	**2**	4 437	4 780		897		3 325	3 839
	**0**	6 442	7 101	454 701	899	2 065	3 870	5 365
**3K5C**	**1**	6 582	7 091		897		3 683	5 338
	**2**	6 578	7 247		899		3 674	5 347
	**0**	4 124	4 947	114 696	933	978	2 965	3 899
**3SU0**	**1**	4 254	4 827		931		2 813	3 970
	**2**	4 164	4 909		925		2 955	3 940
	**0**	3 856	5 800	243 301	976	1 688	3 931	5 359
**4HUS**	**1**	3 710	5 905		976		3 929	5 137
	**2**	3 874	5 679		972		3 880	5 163
	**0**	4 550	4 775	339 672	993	1 380	3 330	3 680
**4NNR**	**1**	4 898	3 835		992		3 250	3 549
	**2**	4 848	4 333		993		3 036	3 562

Table S10. Number of generated ring conformations (full data set).

	Number of Generated Ring Conformations							
	**METHOD NAME**	**MCMM**	**MCMM-ENHANCED**	**MCMM-EXHAUSTIVE**	**PRIME-MCS**	**MD/LLMOD**	**MTLMOD**	**MTLMOD-ENHANCED**
**PDB-CODE**	**SEED**							
**1BXO**	**0**	207	567	1 354	476	137	129	400
	**1**	156	468		458		110	383
	**2**	170	479		475		141	407
**1EHL**	**0**	83	135	196	87	64	92	142
	**1**	106	135		89		116	131
	**2**	80	130		85		89	137
**1ESV**	**0**	186	270	329	196	125	232	258
	**1**	183	266		194		238	260
	**2**	191	267		193		239	239
**1FKD**	**0**	182	1 345	13 389	981	401	337	811
	**1**	114	954		982		284	935
	**2**	116	1 029		979		419	600
**1FKI**	**0**	3 204	6 110	441 066	997	3 256	5 574	6 126
	**1**	3 287	6 149		994		5 467	6 260
	**2**	3 217	6 227		995		5 601	6 195
**1LD8**	**0**	56	56	61	58	35	53	56
	**1**	57	55		56		55	56
	**2**	54	55		57		54	54
**1NMK**	**0**	252	1 345	22 284	959	747	127	952
	**1**	379	1 899		964		181	824
	**2**	305	1 312		958		158	802
**1NSG**	**0**	252	669	21 385	982	782	402	940
	**1**	278	1 280		983		416	863
	**2**	292	998		982		334	741
**1NT1**	**0**	612	1 160	2 860	702	450	524	818
	**1**	587	1 132		699		385	939
	**2**	496	1 200		700		368	891
**1OSF**	**0**	286	1 038	4 069	761	187	258	786
	**1**	216	926		752		235	768
	**2**	216	1 134		769		247	768
**1PKF**	**0**	754	1 963	7 951	906	440	751	1 719
	**1**	678	1 989		907		939	1 606
	**2**	627	1 867		921		860	1 536
**1QY8**	**0**	177	180	186	100	89	177	178
	**1**	176	182		101		179	182
	**2**	175	182		100		180	180
**1S22**	**0**	729	2 790	35 788	923	1 418	493	2 036
	**1**	498	2 916		914		584	1 844
	**2**	418	2 741		913		615	1 760
**1S9D**	**0**	132	142	146	103	53	139	142
	**1**	130	141		104		139	138
	**2**	129	142		104		143	140
**1TPS**	**0**	106	381	1 649	938	130	79	215
	**1**	132	368		936		121	167
	**2**	116	369		936		77	224
**2ASP**	**0**	285	1 724	37 578	999	717	741	1 090
	**1**	129	1 521		996		533	1 336
	**2**	278	1 457		997		603	1 314
**2C6H**	**0**	81	146	178	164	45	112	142
	**1**	84	147		165		109	146
	**2**	80	148		165		108	136
**2DG4**	**0**	313	1 818	23 943	982	687	462	1 208
	**1**	631	1 146		990		576	1 495
	**2**	344	1 400		976		926	1 304
**2E9U**	**0**	616	632	700	372	89	629	644
	**1**	621	629		374		631	649
	**2**	617	631		372		633	646
**2ESA**	**0**	508	1 299	5 076	814	348	405	1 005
	**1**	521	1 315		802		373	1 000
	**2**	503	1 245		808		403	1 050
**2F3F**	**0**	147	688	2 149	618	354	267	543
	**1**	152	587		609		269	545
	**2**	142	612		607		249	522
**2HFK**	**0**	110	107	116	183	81	105	107
	**1**	111	109		181		107	107
	**2**	111	108		184		107	107
**2IWX**	**0**	1 790	2 185	3 623	659	182	1 957	2 157
	**1**	1 743	2 151		665		1 936	2 150
	**2**	1 794	2 224		659		1 970	2 162
**2IYA**	**0**	77	334	903	814	126	118	227
	**1**	64	328		794		64	197
	**2**	75	236		796		93	200

**2J9M**	**0**	54	54	56	58	14	54	54
	**1**	55	54		59		54	55
	**2**	55	54		59		54	54
**2PH8**	**0**	418	521	676	352	274	430	492
	**1**	435	514		358		418	496
	**2**	420	520		358		419	485
**2QZK**	**0**	384	431	510	342	256	359	413
	**1**	393	423		351		332	427
	**2**	378	427		340		345	417
**2WEA**	**0**	19	26	29	56	15	18	23
	**1**	21	24		56		20	26
	**2**	23	26		56		21	26
**2XBK**	**0**	368	1 060	8 643	580	614	739	1 179
	**1**	359	984		568		785	1 185
	**2**	394	885		577		838	1 141
**2XYT**	**0**	60	66	83	48	31	48	71
	**1**	48	74		48		44	65
	**2**	62	66		47		57	67
**3ABA**	**0**	259	938	21 174	948	1 029	349	964
	**1**	255	858		964		465	1 069
	**2**	295	1 065		949		497	1 050
**3BE9**	**0**	59	60	61	51	23	59	60
	**1**	60	60		51		59	60
	**2**	59	60		52		58	60
**3BXR**	**0**	2 405	5 138	189 845	666	774	1 726	3 773
	**1**	2 406	5 037		663		1 552	3 826
	**2**	2 558	5 245		667		1 505	3 776
**3BXS**	**0**	388	533	603	462	126	408	542
	**1**	427	540		454		379	519
	**2**	415	527		450		410	523
**3DV1**	**0**	299	2 422	10 458	906	1 032	684	1 921
	**1**	242	2 136		912		799	1 872
	**2**	311	2 299		912		594	1 777
**3DV5**	**0**	463	2 767	18 504	959	907	738	2 062
	**1**	539	2 778		957		832	2 139
	**2**	415	2 907		961		877	2 073
**3EKS**	**0**	16	19	19	20	12	17	18
	**1**	15	19		19		11	17
	**2**	14	18		19		15	17
**3FRQ**	**0**	48	34	1 105	759	74	68	103
	**1**	48	36		768		105	92
	**2**	56	42		733		89	64
**3I6O**	**0**	58	330	621	355	138	94	252
	**1**	78	290		358		121	256
	**2**	110	292		357		110	280
**3JRX**	**0**	531	1 318	3 767	740	359	559	1 006
	**1**	520	1 311		749		630	1 061
	**2**	558	1 290		732		614	1 077
**3K5C**	**0**	198	837	2 258	591	469	281	770
	**1**	196	848		581		237	704
	**2**	177	905		586		209	766
**3SU0**	**0**	45	196	399	390	98	36	173
	**1**	54	199		407		55	151
	**2**	47	190		397		47	142
**4HUS**	**0**	1 720	4 383	66 840	952	1 485	2 014	3 637
	**1**	1 634	4 483		951		1 952	3 589
	**2**	1 650	4 357		946		2 009	3 563
**4NNR**	**0**	142	1 645	15 214	978	516	436	864
	**1**	199	1 516		981		288	961
	**2**	211	1 765		978		252	1 017

Table S11. Lowest energy conformer generated (kJ mol^{-1}, full data set).

		Lowest energy conformer generated (kJ mol^{-1})					
	**METHOD-NAME**	**MCMM**	**MCMM-ENHANCED**	**MCMM-EXHAUSTIVE**	**MD/LLMOD**	**MTLMOD**	**MTLMOD-ENHANCED**
**PDB-CODE**	**SEED**						
**1BXO**	**0**	-750.1	-747.6	-755.8	-749.6	-750.0	-753.6
	**1**	-751.4	-755.7			-753.6	-755.8
	**2**	-752.1	-751.7			-752.1	-750.9
**1EHL**	**0**	-601.6	-601.6	-601.6	-601.6	-601.6	-601.6
	**1**	-601.6	-601.6			-601.6	-601.6
	**2**	-601.6	-601.6			-601.6	-601.6
**1ESV**	**0**	-505.6	-505.6	-505.6	-505.6	-505.6	-505.6
	**1**	-505.6	-505.6			-505.6	-505.6
	**2**	-505.6	-505.6			-505.6	-505.6
**1FKD**	**0**	155.1	148.4	131.2	132.8	150.7	149.5
	**1**	145.8	140.5			150.6	153.2
	**2**	148.3	138.8			159.0	142.0
**1FKI**	**0**	210.8	211.2	207.6	210.8	210.2	211.4
	**1**	212.7	212.7			210.1	211.6
	**2**	208.7	212.7			213.2	209.2
**1LD8**	**0**	218.1	218.1	218.1	218.1	218.1	218.1
	**1**	218.1	218.1			218.1	218.1
	**2**	218.1	218.1			218.1	218.1
**1NMK**	**0**	-413.5	-410.9	-414.2	-409.4	-404.1	-410.7
	**1**	-410.7	-401.1			-405.6	-410.2
	**2**	-404.2	-412.1			-401.5	-411.1
**1NSG**	**0**	45.7	42.3	38.3	60.8	42.5	46.9
	**1**	45.4	52.9			45.7	47.7
	**2**	45.4	45.7			47.7	42.4
**1NT1**	**0**	-294.6	-294.6	-294.7	-294.6	-294.6	-294.6
	**1**	-294.6	-294.6			-294.6	-294.6
	**2**	-294.6	-294.6			-294.6	-294.6
**1OSF**	**0**	-34.3	-35.6	-37.9	-35.5	-33.0	-35.5
	**1**	-33.0	-37.9			-33.8	-37.1
	**2**	-35.6	-33.1			-35.5	-35.1
**1PKF**	**0**	-331.0	-329.1	-331.0	-331.0	-326.0	-328.9
	**1**	-330.6	-329.2			-326.1	-331.0
	**2**	-324.6	-330.6			-330.6	-330.6
**1QY8**	**0**	-396.1	-396.1	-396.1	-396.1	-396.1	-396.1
	**1**	-396.1	-396.1			-396.1	-396.1
	**2**	-396.1	-396.1			-396.1	-396.1
**1S22**	**0**	-435.3	-435.4	-441.4	-441.4	-435.2	-437.3
	**1**	-433.1	-434.7			-436.9	-440.4
	**2**	-436.9	-437.4			-436.2	-439.8
**1S9D**	**0**	-187.0	-187.0	-187.0	-187.0	-187.0	-187.0
	**1**	-187.0	-187.0			-187.0	-187.0
	**2**	-187.0	-187.0			-187.0	-187.0
**1TPS**	**0**	-909.3	-903.7	-924.3	-913.4	-913.0	-911.5
	**1**	-904.4	-910.1			-911.8	-911.3
	**2**	-902.6	-908.0			-923.6	-911.0
**2ASP**	**0**	-569.6	-564.1	-586.3	-585.3	-575.9	-578.5
	**1**	-582.1	-568.6			-576.2	-575.3
	**2**	-577.2	-570.5			-581.2	-570.5
**2C6H**	**0**	-18.2	-18.2	-18.3	-6.3	-18.2	-18.2
	**1**	-18.2	-18.2			-18.2	-18.2
	**2**	-18.2	-18.2			-18.2	-18.2
**2DG4**	**0**	86.4	102.2	79.5	94.6	88.2	93.4
	**1**	97.9	92.0			88.2	103.4
	**2**	82.6	96.8			95.7	97.7
**2E9U**	**0**	-230.5	-230.5	-230.5	-230.5	-230.5	-230.5
	**1**	-230.5	-230.5			-230.5	-230.5
	**2**	-230.5	-230.5			-230.5	-230.5
**2ESA**	**0**	-147.2	-147.2	-147.2	-147.2	-144.6	-146.1
	**1**	-146.0	-146.1			-144.6	-147.2
	**2**	-147.2	-147.2			-147.2	-147.2
**2F3F**	**0**	-277.2	-281.1	-286.0	-285.9	-278.6	-279.0
	**1**	-280.1	-279.0			-280.3	-281.1
	**2**	-280.0	-280.0			-286.0	-280.1
**2HFK**	**0**	-84.6	-84.6	-84.6	-84.5	-84.5	-84.6
	**1**	-84.5	-84.6			-84.6	-84.6
	**2**	-84.5	-84.5			-84.6	-84.5

**2IWX**	**0**	-377.3	-377.3	-377.3	-377.3	-377.3	-377.3
	**1**	-377.3	-377.3			-377.3	-377.3
	**2**	-377.3	-377.3			-377.3	-377.3
**2IYA**	**0**	-133.8	-127.8	-133.9	-111.9	-133.8	-133.8
	**1**	-133.8	-128.8			-114.1	-133.8
	**2**	-133.8	-133.8			-117.3	-133.8
**2J9M**	**0**	-847.0	-847.0	-847.0	-847.0	-847.0	-847.0
	**1**	-847.0	-847.0			-847.0	-847.0
	**2**	-847.0	-847.0			-847.0	-847.0
**2PH8**	**0**	-404.1	-404.1	-404.1	-404.1	-404.1	-404.1
	**1**	-404.1	-404.1			-404.1	-404.1
	**2**	-404.1	-404.1			-404.1	-404.1
**2QZK**	**0**	-102.5	-102.5	-102.6	-102.5	-102.5	-102.5
	**1**	-102.6	-102.6			-102.5	-102.6
	**2**	-102.6	-102.6			-102.6	-102.5
**2WEA**	**0**	-315.9	-315.9	-315.9	-315.9	-315.9	-315.9
	**1**	-315.9	-315.9			-315.9	-315.9
	**2**	-315.9	-315.9			-315.9	-315.9
**2XBK**	**0**	-1188.6	-1189.2	-1189.2	-1189.2	-1189.2	-1189.2
	**1**	-1189.2	-1189.2			-1189.2	-1189.2
	**2**	-1188.1	-1189.2			-1189.2	-1189.2
**2XYT**	**0**	240.6	241.0	240.3	241.0	241.0	240.6
	**1**	241.0	240.6			241.0	241.0
	**2**	241.0	241.0			240.6	241.0
**3ABA**	**0**	-763.8	-761.2	-765.4	-761.4	-764.8	-762.3
	**1**	-765.4	-764.8			-761.3	-762.3
	**2**	-764.8	-757.6			-760.0	-759.5
**3BE9**	**0**	-630.2	-630.2	-630.2	-630.1	-630.2	-630.2
	**1**	-630.1	-630.2			-630.1	-630.2
	**2**	-630.2	-630.1			-630.1	-630.2
**3BXR**	**0**	-597.9	-597.9	-597.9	-597.9	-596.9	-597.9
	**1**	-597.7	-596.8			-587.6	-596.6
	**2**	-591.8	-597.6			-597.7	-594.2
**3BXS**	**0**	-331.4	-331.4	-331.4	-331.4	-331.4	-331.4
	**1**	-331.4	-331.4			-331.4	-331.4
	**2**	-331.4	-331.4			-331.4	-331.4
**3DV1**	**0**	-324.5	-322.7	-328.0	-323.2	-328.0	-322.6
	**1**	-328.0	-328.0			-321.7	-320.9
	**2**	-324.5	-324.2			-323.2	-322.6
**3DV5**	**0**	-93.4	-92.5	-96.4	-96.3	-94.7	-94.7
	**1**	-88.8	-93.4			-94.9	-94.9
	**2**	-96.4	-94.9			-93.9	-96.2
**3EKS**	**0**	-158.8	-158.8	-158.8	-158.8	-158.8	-158.8
	**1**	-158.8	-158.8			-158.8	-158.8
	**2**	-158.8	-158.8			-158.8	-158.8
**3FRQ**	**0**	-104.8	-104.7	-104.8	-100.8	-104.8	-104.8
	**1**	-104.7	-104.8			-104.8	-104.8
	**2**	-104.8	-104.8			-104.8	-104.8
**3I6O**	**0**	-334.1	-334.5	-338.2	-338.2	-336.3	-338.3
	**1**	-333.2	-338.3			-334.6	-336.8
	**2**	-326.5	-336.9			-338.2	-336.9
**3JRX**	**0**	-21.3	-24.3	-24.3	-24.3	-24.3	-24.3
	**1**	-13.4	-24.3			-24.3	-20.5
	**2**	-9.4	-24.3			-21.3	-24.3
**3K5C**	**0**	-99.5	-99.1	-100.4	-96.5	-96.2	-96.6
	**1**	-96.2	-99.1			-96.3	-100.4
	**2**	-96.2	-96.5			-96.3	-98.3
**3SU0**	**0**	-710.5	-716.4	-716.4	-716.4	-710.5	-715.3
	**1**	-712.0	-716.4			-710.3	-715.3
	**2**	-710.5	-716.4			-714.4	-715.5
**4HUS**	**0**	-415.6	-415.1	-415.7	-415.7	-415.7	-412.2
	**1**	-415.7	-414.0			-414.8	-415.7
	**2**	-412.2	-415.6			-415.7	-415.6
**4NNR**	**0**	104.9	115.3	103.0	107.8	122.5	109.0
	**1**	106.7	110.0			120.1	108.5
	**2**	107.1	115.3			108.1	113.5

Table S12. Elapsed computational time in minutes (full data set).

		Elapsed computational time in minutes.						
	METHOD NAME	MCMM	MCMM-ENHANCED	MCMM-EXHAUSTIVE	PRIME-MCS	MD/LLMOD	MTLMOD	MTLMOD-ENHANCED
PDB CODE	SEED							
1BXO	0	84.0	106.1	33 065.0	220.9	112.8	83.2	101.5
	1	91.1	107.9		220.5		90.8	95.0
	2	95.2	108.5		220.6		101.5	98.8
1EHL	0	39.4	166.7	8 228.0	216.0	40.8	166.0	119.5
	1	70.3	120.4		215.5		147.6	135.3
	2	101.0	114.5		215.0		135.9	126.0
1ESV	0	15.5	27.4	2 211.0	214.7	36.0	22.5	26.1
	1	19.9	32.1		214.3		26.9	30.5
	2	20.1	32.4		214.0		26.7	30.5
1FKD	0	130.2	176.9	33 978.0	229.8	183.0	165.1	169.2
	1	142.0	179.8		229.3		107.0	142.8
	2	140.4	183.3		229.2		131.8	149.3
1FKI	0	32.3	55.6	33 164.0	215.8	67.2	45.3	51.1
	1	28.7	46.3		215.3		40.5	44.4
	2	28.5	47.2		215.1		40.3	44.9
1LD8	0	18.8	19.3	1 969.0	213.2	28.8	24.4	18.0
	1	15.4	19.2		212.9		22.0	17.7
	2	15.7	19.1		213.4		22.1	17.7
1NMK	0	107.8	142.3	35 838.0	224.6	149.4	115.8	127.0
	1	94.0	124.4		224.5		99.6	110.4
	2	90.2	124.9		224.7		101.0	109.1
1NSG	0	152.8	252.7	32 385.0	236.8	241.2	193.0	207.2
	1	151.4	222.7		237.1		174.7	191.9
	2	143.5	221.6		236.9		195.3	148.2
1NT1	0	35.0	55.0	5 114.0	217.3	63.0	42.9	48.1
	1	30.6	49.0		216.8		40.6	44.2
	2	30.9	50.5		216.4		39.8	44.7
1OSF	0	136.3	123.1	33 503.0	229.6	107.4	99.4	124.5
	1	71.8	120.2		229.4		79.4	90.4
	2	118.7	111.3		229.8		83.1	101.7
1PKF	0	41.9	60.1	23 542.0	216.4	71.4	49.7	55.6
	1	39.7	54.9		216.6		45.3	50.7
	2	40.3	55.2		216.4		45.3	50.0
1QY8	0	14.2	19.3	1 271.0	213.1	22.2	16.8	18.4
	1	13.7	18.8		212.9		9.9	10.0
	2	13.7	18.8		212.9		10.1	10.9
1S22	0	81.9	112.8	41 417.0	227.0	132.0	87.5	97.2
	1	63.2	95.4		227.1		72.5	84.9
	2	63.0	96.6		227.1		75.2	85.2
1S9D	0	7.2	9.4	1 166.0	212.9	25.8	9.2	9.5
	1	9.3	12.3		212.9		12.3	12.6
	2	9.5	12.3		213.7		12.4	13.1
1TPS	0	209.8	320.4	47 026.0	254.5	301.8	217.8	237.8
	1	181.7	299.7		254.1		209.1	173.1
	2	176.3	275.6		253.7		168.1	179.8
2ASP	0	169.7	236.1	33 458.0	240.7	248.4	197.6	215.5
	1	176.2	239.1		240.2		184.4	215.0
	2	123.0	220.1		240.3		222.1	213.6
2C6H	0	45.7	56.5	3 875.0	219.6	61.2	46.3	51.2
	1	40.3	51.3		219.2		43.3	46.8
	2	39.8	62.9		218.8		45.8	47.3
2DG4	0	148.5	220.3	27 987.0	238.9	216.0	195.0	184.3
	1	138.4	214.3		238.8		167.5	197.1
	2	150.2	210.2		238.5		159.9	176.4
2E9U	0	12.5	17.8	1 165.0	215.5	24.0	16.9	16.9
	1	13.0	17.4		215.0		8.2	18.0
	2	12.8	17.4		214.6		8.6	16.3
2ESA	0	50.1	79.5	11 628.0	219.5	71.4	55.1	63.4
	1	43.0	58.1		219.2		46.9	50.8
	2	41.2	67.3		218.7		44.2	52.3
2F3F	0	55.7	66.9	19 985.0	219.8	80.4	58.0	64.9
	1	47.9	53.6		219.4		48.0	53.3
	2	46.2	52.9		219.8		48.7	52.5
2HFK	0	15.1	21.4	1 111.0	218.3	23.4	18.3	20.3
	1	12.4	18.2		217.7		15.5	17.9
	2	12.5	18.4		217.2		15.8	17.4
2IWX	0	19.5	25.8	2 131.0	217.3	26.4	22.6	24.8
	1	17.0	21.3		216.9		12.9	20.4
	2	16.8	20.9		216.4		13.1	13.1
2IYA	0	106.9	127.5	17 094.0	222.9	122.4	109.1	117.0
	1	90.9	107.4		222.7		100.8	99.3
	2	91.0	118.0		223.1		93.8	99.0

**2J9M**	**0**	5.8	7.5	744.0	214.7	15.0	5.7	6.7
	**1**	6.7	9.0		214.3		7.6	8.9
	**2**	6.9	9.0		213.9		7.4	8.6
**2PH8**	**0**	35.1	49.2	3 758.0	215.3	61.8	36.5	44.3
	**1**	34.3	42.7		214.8		31.0	38.2
	**2**	32.8	42.3		214.6		31.7	39.1
**2QZK**	**0**	47.6	64.9	4 290.0	214.6	68.4	48.7	54.2
	**1**	36.4	54.1		214.4		40.6	34.2
	**2**	38.0	49.9		214.6		42.7	50.5
**2WEA**	**0**	49.2	57.7	4 111.0	220.7	72.6	51.9	54.2
	**1**	46.9	54.3		220.3		47.2	53.3
	**2**	47.4	55.7		220.7		49.0	52.1
**2XBK**	**0**	62.3	92.9	13 799.0	222.5	102.0	74.3	88.1
	**1**	58.6	78.1		223.1		68.2	73.6
	**2**	64.5	76.5		222.7		72.1	72.1
**2XYT**	**0**	49.5	77.5	4 719.0	220.5	67.2	59.7	65.7
	**1**	48.3	66.1		220.1		56.9	58.8
	**2**	47.3	67.2		219.7		54.4	60.0
**3ABA**	**0**	80.4	113.5	17 731.0	233.0	129.6	87.8	103.9
	**1**	64.2	87.6		232.7		64.7	74.9
	**2**	43.5	85.9		232.7		65.1	76.9
**3BE9**	**0**	23.3	32.0	1 912.0	218.3	33.0	28.2	34.6
	**1**	17.3	22.1		218.0		22.7	23.4
	**2**	18.0	23.3		217.9		24.4	23.8
**3BXR**	**0**	94.8	107.7	35 972.0	222.0	126.6	93.3	98.1
	**1**	71.6	100.0		221.6		68.2	92.3
	**2**	68.9	105.0		221.8		68.2	92.0
**3BXS**	**0**	20.7	27.6	1 746.0	214.1	34.2	23.9	26.2
	**1**	17.6	24.9		213.7		20.4	21.9
	**2**	18.1	22.5		213.5		20.0	21.5
**3DV1**	**0**	61.2	65.8	40 945.0	218.9	97.2	58.9	62.4
	**1**	46.8	50.7		218.5		34.0	49.2
	**2**	46.8	49.3		219.1		51.9	49.5
**3DV5**	**0**	68.9	76.1	35 417.0	233.3	108.0	70.6	73.3
	**1**	65.9	71.0		233.2		65.1	70.1
	**2**	65.1	71.3		233.5		65.3	69.2
**3EKS**	**0**	40.7	48.8	6 661.0	219.5	58.8	45.6	47.9
	**1**	37.8	44.4		219.2		42.9	43.6
	**2**	37.4	44.5		218.8		43.5	44.2
**3FRQ**	**0**	98.9	96.8	22 159.0	224.9	133.8	106.1	110.5
	**1**	98.3	93.9		224.7		104.1	106.1
	**2**	103.9	97.2		224.9		105.6	103.9
**3I6O**	**0**	66.9	76.2	28 256.0	220.7	114.0	69.2	71.6
	**1**	69.4	73.1		220.3		66.8	73.2
	**2**	63.7	71.8		220.3		43.4	69.1
**3JRX**	**0**	46.5	58.1	13 657.0	217.6	66.0	50.2	53.4
	**1**	39.2	55.6		217.2		46.9	52.9
	**2**	41.9	56.4		216.8		47.1	51.1
**3K5C**	**0**	122.0	114.5	42 172.0	237.9	109.8	100.2	99.4
	**1**	145.1	129.1		237.6		106.3	122.9
	**2**	149.7	130.3		237.2		104.5	100.6
**3SU0**	**0**	887.2	1 095.3	111 171.0	220.9	96.0	1 083.0	992.6
	**1**	1 078.1	986.5		220.6		1 171.2	974.6
	**2**	1 004.9	904.8		222.1		1 144.8	970.5
**4HUS**	**0**	23.9	41.6	42 175.0	222.2	55.2	31.6	36.3
	**1**	36.9	56.1		221.9		35.5	30.9
	**2**	35.0	55.6		221.5		35.3	42.0
**4NNR**	**0**	179.5	161.2	41 320.0	234.0	157.2	123.9	151.7
	**1**	111.3	144.3		233.6		132.1	131.6
	**2**	137.1	155.4		233.2		112.4	120.5

Table S13. Conformer with the lowest heavy atom Root Mean Square Deviation (RMSD) in Ångtröm to the X-ray$_{ppw}$ conformation (full data set).

		Heavy-Atom RMSD								
	**METHOD NAME**	**ENERGY MINIMIZED X-RAY**	**MCMM**	**MCMM-EXHAUSTIVE**	**MCMM-ENHANCED**	**PRIME-MCS**	**MD/LLMOD**	**MTLMOD**	**MTLMOD-ENHANCED**	**STARTING CONFORMER**
**PDB CODE**	**SEED**									
	**0**	1.34	1.00	0.59	0.80	1.31	0.81	0.79	0.95	4.71
**1BXO**	**1**		0.92		0.81	1.31		0.74	1.02	
	**2**		0.60		0.84	1.31		0.87	0.81	
	**0**	0.17	0.15	0.14	0.15	0.76	0.16	0.15	0.15	2.30
**1EHL**	**1**		0.15		0.15	0.76		0.16	0.16	
	**2**		0.15		0.16	0.79		0.16	0.16	
	**0**	0.23	0.22	0.22	0.22	0.56	0.44	0.22	0.22	2.75
**1ESV**	**1**		0.23		0.22	0.56		0.22	0.22	
	**2**		0.22		0.22	0.56		0.22	0.22	
	**0**	0.29	0.30	0.30	0.74	1.03	1.03	0.43	0.76	3.72
**1FKD**	**1**		0.29		0.59	1.01		0.92	0.66	
	**2**		0.29		1.60	1.01		1.51	1.70	
	**0**	0.25	0.52	0.42	0.64	0.66	0.56	0.94	0.56	3.39
**1FKI**	**1**		0.85		0.70	0.71		0.59	0.76	
	**2**		0.65		0.68	0.57		0.71	0.80	
	**0**	0.71	0.54	0.54	0.54	0.53	0.53	0.54	0.54	2.79
**1LD8**	**1**		0.54		0.54	0.53		0.54	0.54	
	**2**		0.54		0.54	0.53		0.54	0.54	
	**0**	0.40	0.91	0.43	0.87	1.28	0.81	1.15	1.20	4.49
**1NMK**	**1**		0.68		0.91	1.28		1.58	1.02	
	**2**		1.10		1.04	1.28		1.83	1.23	
	**0**	0.32	0.33	0.30	0.84	1.39	1.65	0.32	0.38	3.69
**1NSG**	**1**		0.30		0.37	1.57		0.31	0.67	
	**2**		0.31		0.34	1.57		0.38	0.33	
	**0**	0.57	0.57	0.38	0.57	0.65	0.57	0.57	0.57	4.29
**1NT1**	**1**		0.57		0.57	0.65		0.57	0.57	
	**2**		0.57		0.57	0.65		0.57	0.57	
	**0**	0.67	0.79	0.47	1.21	1.38	2.18	1.76	1.19	4.43
**1OSF**	**1**		1.61		0.54	1.38		2.07	0.98	
	**2**		1.93		0.57	1.38		2.10	1.03	
	**0**	0.35	0.74	0.32	0.41	1.06	0.74	0.92	0.55	3.68
**1PKF**	**1**		1.25		0.48	1.14		0.41	0.59	
	**2**		1.20		0.41	1.06		0.75	0.48	
	**0**	0.35	0.07	0.07	0.07	0.41	0.25	0.07	0.07	3.18
**1QY8**	**1**		0.07		0.07	0.41		0.07	0.07	
	**2**		0.07		0.07	0.41		0.07	0.07	
	**0**	0.63	1.88	0.59	1.33	1.19	0.91	3.31	0.73	4.80
**1S22**	**1**		2.05		0.97	1.19		2.95	1.45	
	**2**		2.40		0.98	1.19		3.00	1.78	
	**0**	0.21	0.21	0.21	0.21	0.55	0.21	0.21	0.21	1.77
**1S9D**	**1**		0.21		0.21	0.55		0.21	0.21	
	**2**		0.21		0.21	0.55		0.21	0.21	
	**0**	1.36	2.34	1.22	1.78	2.30	2.01	2.79	1.78	5.73
**1TPS**	**1**		2.30		1.75	2.30		2.00	2.12	
	**2**		1.82		1.83	2.30		2.54	2.06	
	**0**	0.37	1.88	0.53	1.74	2.49	1.57	1.86	1.37	5.94
**2ASP**	**1**		0.56		1.02	2.38		1.44	1.52	
	**2**		1.20		1.48	2.38		1.98	1.17	
	**0**	1.12	0.67	0.67	0.90	0.82	1.10	0.67	0.83	3.22
**2C6H**	**1**		0.67		0.70	0.82		0.67	0.68	
	**2**		0.67		0.78	0.82		0.74	0.85	
	**0**	0.53	0.47	0.44	1.13	1.86	1.24	0.53	0.61	4.94
**2DG4**	**1**		0.61		0.67	1.83		0.57	1.36	
	**2**		0.35		1.13	1.86		1.45	0.80	
	**0**	0.93	0.37	0.37	0.37	0.37	0.62	0.37	0.37	2.24
**2E9U**	**1**		0.37		0.37	0.37		0.37	0.37	
	**2**		0.37		0.37	0.37		0.37	0.37	
	**0**	0.22	0.94	0.20	0.20	0.24	0.20	1.21	0.33	3.76
**2ESA**	**1**		1.83		0.21	0.24		0.94	0.51	
	**2**		1.08		0.20	0.24		0.97	0.79	
	**0**	0.93	0.92	0.74	1.00	0.89	1.13	0.94	0.74	4.63
**2F3F**	**1**		1.04		1.21	0.89		0.82	1.08	
	**2**		1.10		1.07	0.89		1.23	1.18	
	**0**	0.13	0.13	0.13	0.13	0.52	0.13	0.13	0.13	1.95
**2HFK**	**1**		0.13		0.13	0.52		0.13	0.13	
	**2**		0.13		0.13	0.52		0.13	0.13	
	**0**	0.20	0.18	0.18	0.18	0.41	0.54	0.18	0.18	2.89
**2IWX**	**1**		0.18		0.32	0.41		0.18	0.18	
	**2**		0.20		0.18	0.41		0.18	0.18	
	**0**	0.37	0.48	0.43	0.53	1.18	0.95	0.44	0.70	4.79
**2IYA**	**1**		0.49		0.49	1.24		1.69	0.44	
	**2**		0.49		0.49	1.24		0.98	0.68	
	**0**	0.13	0.78	0.78	0.78	0.78	0.35	0.78	0.78	1.42
**2J9M**	**1**		0.78		0.78	0.78		0.78	0.78	
	**2**		0.78		0.78	0.78		0.78	0.78	
	**0**	0.60	0.57	0.57	0.57	0.88	0.68	0.57	0.57	3.17
**2PH8**	**1**		0.57		0.57	0.80		0.57	0.57	
	**2**		0.57		0.57	0.80		0.57	0.57	
	**0**	0.79	0.90	0.78	0.91	0.96	0.96	0.96	0.78	3.67
**2QZK**	**1**		0.96		0.82	0.95		0.90	0.95	
	**2**		0.91		0.91	1.05		0.78	0.78	
	**0**	0.39	0.88	0.80	0.48	1.59	1.32	0.65	0.48	4.43
**2WEA**	**1**		0.40		0.40	1.56		0.49	0.67	
	**2**		0.48		0.48	1.56		0.65	0.76	
	**0**	0.68	0.80	0.59	0.76	0.76	0.73	0.60	0.60	3.30
**2XBK**	**1**		0.79		0.59	0.77		0.61	0.59	
	**2**		0.86		0.64	0.78		0.72	0.60	
	**0**	0.23	0.22	0.22	0.22	0.23	0.76	0.23	0.35	3.15
**2XYT**	**1**		0.23		0.23	0.23		0.23	0.22	
	**2**		0.24		0.22	0.23		0.25	0.22	
	**0**	0.43	0.44	0.41	0.61	1.34	0.69	0.41	0.46	4.12
**3ABA**	**1**		0.42		0.59	1.27		0.40	0.44	
	**2**		0.42		0.46	1.26		0.41	0.41	
	**0**	0.40	0.56	0.54	0.56	0.78	0.56	0.56	0.56	1.63
**3BE9**	**1**		0.56		0.56	0.78		0.56	0.40	
	**2**		0.56		0.56	0.78		0.56	0.56	
	**0**	0.58	0.79	0.56	0.69	1.61	1.00	0.90	0.87	4.87
**3BXR**	**1**		0.89		0.69	1.61		0.74	0.79	
	**2**		0.75		0.80	1.61		0.96	0.70	
	**0**	0.38	0.38	0.38	0.38	0.55	0.55	0.38	0.38	1.97
**3BXS A**	**1**		0.38		0.38	0.54		0.38	0.38	
	**2**		0.38		0.38	0.54		0.38	0.38	

**3BXS B**	**0**	0.38	0.37	0.37	0.37	0.38	0.47	0.37	0.37	1.81
	**1**		0.37		0.37	0.38		0.37	0.37	
	**2**		0.37		0.37	0.38		0.37	0.37	
**3DV1**	**0**	0.98	1.03	0.79	0.84	1.28	0.88	1.00	0.90	4.18
	**1**		0.90		0.83	1.28		0.84	0.84	
	**2**		0.90		0.85	1.28		0.94	1.01	
**3DV5**	**0**	0.72	0.91	0.77	0.91	1.44	1.15	1.08	1.13	4.45
	**1**		0.89		0.91	1.44		1.16	1.00	
	**2**		1.05		0.99	1.44		0.91	1.13	
**3EKS**	**0**	0.26	0.26	0.25	0.26	0.74	0.25	0.25	0.26	1.97
	**1**		0.26		0.25	0.74		0.25	0.25	
	**2**		0.26		0.25	0.74		0.25	0.26	
**3FRQ**	**0**	0.49	0.48	0.43	0.50	1.43	1.34	0.48	0.48	3.22
	**1**		0.46		0.43	1.07		0.50	0.48	
	**2**		0.43		0.50	1.43		0.50	0.56	
**3I6O**	**0**	1.03	0.76	0.66	0.86	1.66	1.56	0.82	0.80	5.10
	**1**		0.76		0.82	1.85		0.90	0.86	
	**2**		1.08		0.77	1.66		0.80	0.91	
**3JRX**	**0**	0.23	0.27	0.26	0.23	0.53	0.54	0.26	0.35	3.11
	**1**		0.67		0.27	0.43		0.50	0.26	
	**2**		0.52		0.35	0.53		0.52	0.26	
**3K5C**	**0**	0.76	0.70	0.70	0.80	1.27	0.97	0.95	0.81	4.39
	**1**		0.83		0.70	1.27		0.95	0.81	
	**2**		0.82		0.86	1.27		0.89	0.89	
**3SU0**	**0**	1.12	0.78	0.71	0.91	1.56	0.75	0.91	0.95	4.18
	**1**		0.88		0.94	1.51		1.18	1.07	
	**2**		0.74		1.05	1.56		1.24	0.93	
**4HUS**	**0**	0.64	0.61	0.61	0.77	1.19	0.74	0.61	0.64	3.25
	**1**		0.72		0.73	1.19		0.61	0.65	
	**2**		0.65		0.70	1.19		0.61	0.65	
**4NNR**	**0**	0.55	1.56	0.49	1.32	0.74	2.44	1.34	1.59	4.76
	**1**		2.54		1.12	0.74		1.39	1.62	
	**2**		1.58		1.05	1.02		2.45	0.84	

Table S14. Mean values of the lowest heavy atom Root Mean Square Deviation (RMSD) in Ångtröm to the X-ray$_{ppw}$ conformation (full data set).

				Mean Heavy-Atom RMSD					
**METHOD NAME**	**ENERGY MINIMIZED X-RAY**	**MCMM**	**MCMM-EXHAUSTIVE**	**MCMM-ENHANCED**	**PRIME-MCS**	**MD/LLMOD**	**MTLMOD**	**MTLMOD-ENHANCED**	**STARTING CONFORMER**
**PDB CODE**									
**1BXO**	1.34	0.84	0.59	0.82	1.31	0.81	0.80	0.93	4.71
**1EHL**	0.17	0.15	0.14	0.15	0.77	0.16	0.16	0.16	2.30
**1ESV**	0.23	0.22	0.22	0.22	0.56	0.44	0.22	0.22	2.75
**1FKD**	0.29	0.29	0.30	0.98	1.02	1.03	0.95	1.04	3.72
**1FKI**	0.25	0.67	0.42	0.67	0.65	0.56	0.75	0.71	3.39
**1LD8**	0.71	0.54	0.54	0.54	0.53	0.53	0.54	0.54	2.79
**1NMK**	0.40	0.90	0.43	0.94	1.28	0.81	1.52	1.15	4.49
**1NSG**	0.32	0.31	0.30	0.52	1.51	1.65	0.34	0.46	3.69
**1NT1**	0.57	0.57	0.38	0.57	0.65	0.57	0.57	0.57	4.29
**1OSF**	0.67	1.44	0.47	0.77	1.38	2.18	1.98	1.07	4.43
**1PKF**	0.35	1.06	0.32	0.43	1.09	0.74	0.69	0.54	3.68
**1QY8**	0.35	0.07	0.07	0.07	0.41	0.25	0.07	0.07	3.18
**1S22**	0.63	2.11	0.59	1.09	1.19	0.91	3.08	1.32	4.80
**1S9D**	0.21	0.21	0.21	0.21	0.55	0.21	0.21	0.21	1.77
**1TPS**	1.36	2.15	1.22	1.79	2.30	2.01	2.44	1.99	5.73
**2ASP**	0.37	1.21	0.53	1.41	2.42	1.57	1.76	1.35	5.94
**2C6H**	1.12	0.67	0.67	0.79	0.82	1.10	0.69	0.79	3.22
**2DG4**	0.53	0.48	0.44	0.98	1.85	1.24	0.85	0.92	4.94
**2E9U**	0.93	0.37	0.37	0.37	0.37	0.62	0.37	0.37	2.24
**2ESA**	0.22	1.28	0.20	0.20	0.24	0.20	1.04	0.54	3.76
**2F3F**	0.93	1.02	0.74	1.09	0.89	1.13	1.00	1.00	4.63
**2HFK**	0.13	0.13	0.13	0.13	0.52	0.13	0.13	0.13	1.95
**2IWX**	0.20	0.19	0.18	0.23	0.41	0.54	0.18	0.18	2.89
**2IYA**	0.37	0.49	0.43	0.50	1.22	0.95	1.04	0.61	4.79
**2J9M**	0.13	0.78	0.78	0.78	0.78	0.35	0.78	0.78	1.42
**2PH8**	0.60	0.57	0.57	0.57	0.83	0.68	0.57	0.57	3.17
**2QZK**	0.79	0.92	0.78	0.88	0.99	0.96	0.88	0.84	3.67
**2WEA**	0.39	0.59	0.80	0.45	1.57	1.32	0.60	0.64	4.43
**2XBK**	0.68	0.82	0.59	0.66	0.77	0.73	0.64	0.60	3.30
**2XYT**	0.23	0.23	0.22	0.22	0.23	0.76	0.24	0.26	3.15
**3ABA**	0.43	0.43	0.41	0.55	1.29	0.69	0.41	0.44	4.12
**3BE9**	0.40	0.56	0.54	0.56	0.78	0.56	0.56	0.51	1.63
**3BXR**	0.58	0.81	0.56	0.73	1.61	1.00	0.87	0.79	4.87
**3BXSA**	0.38	0.38	0.38	0.38	0.54	0.55	0.38	0.38	1.97
**3BXSB**	0.38	0.37	0.37	0.37	0.38	0.47	0.37	0.37	1.81
**3DV1**	0.98	0.94	0.79	0.84	1.28	0.88	0.93	0.92	4.18
**3DV5**	0.72	0.95	0.77	0.94	1.44	1.15	1.05	1.09	4.45
**3EKS**	0.26	0.26	0.25	0.25	0.74	0.25	0.25	0.26	1.97
**3FRQ**	0.49	0.46	0.43	0.48	1.31	1.34	0.49	0.51	3.22
**3I6O**	1.03	0.87	0.66	0.82	1.72	1.56	0.84	0.86	5.10
**3JRX**	0.23	0.49	0.26	0.28	0.50	0.54	0.43	0.29	3.11
**3K5C**	0.76	0.78	0.70	0.79	1.27	0.97	0.93	0.84	4.39
**3SU0**	1.12	0.80	0.71	0.97	1.54	0.75	1.11	0.98	4.18
**4HUS**	0.64	0.66	0.61	0.73	1.19	0.74	0.61	0.65	3.25
**4NNR**	0.55	1.89	0.49	1.16	0.83	2.44	1.73	1.35	4.76

Table S15. Conformer with the lowest Root Mean Square Deviation ($RMSD_{RING}$) in Ångtröm to the X-ray_{ppw} ring conformation (full data set).

	METHOD NAME	ENERGY MINIMIZED X-RAY	MCMM	MCMM-EXHAUSTIVE	MCMM-ENHANCED	PRIME-MCS	MD/LLMOD	MTLMOD	MTLMOD-ENHANCED	STARTING CONFORMER
		Ring Atom $RMSD_{RING}$								
PDB CODE	SEED									
**1BXO**	**0**	0.28	0.19	0.18	0.2	0.3	0.19	0.21	0.19	0.64
	**1**		0.2		0.19	0.3		0.19	0.19	
	**2**		0.19		0.19	0.3		0.19	0.19	
**1EHL**	**0**	0.12	0.12	0.1	0.12	0.49	0.13	0.12	0.12	0.95
	**1**		0.12		0.12	0.49		0.12	0.12	
	**2**		0.12		0.12	0.48		0.12	0.12	
**1ESV**	**0**	0.1	0.1	0.1	0.1	0.39	0.16	0.1	0.1	1.22
	**1**		0.1		0.1	0.39		0.1	0.1	
	**2**		0.1		0.1	0.49		0.1	0.1	
**1FKD**	**0**	0.1	0.08	0.08	0.44	0.59	0.5	0.1	0.09	0.44
	**1**		0.08		0.22	0.51		0.37	0.14	
	**2**		0.08		0.61	0.59		0.44	0.44	
**1FKI**	**0**	0.16	0.53	0.17	0.67	0.66	0.63	0.7	0.48	2.26
	**1**		0.64		0.58	0.71		0.64	0.68	
	**2**		0.66		0.66	0.53		0.66	0.54	
**1LD8**	**0**	0.38	0.25	0.25	0.25	0.25	0.25	0.25	0.25	1.12
	**1**		0.25		0.25	0.25		0.25	0.25	
	**2**		0.25		0.25	0.25		0.25	0.25	
**1NMK**	**0**	0.09	0.24	0.08	0.25	0.49	0.19	0.57	0.25	1.66
	**1**		0.1		0.46	0.49		0.69	0.47	
	**2**		0.56		0.27	0.43		1.03	0.52	
**1NSG**	**0**	0.25	0.17	0.17	0.2	0.82	0.47	0.19	0.17	1.06
	**1**		0.16		0.22	0.82		0.14	0.24	
	**2**		0.17		0.17	0.82		0.17	0.2	
**1NT1**	**0**	0.36	0.35	0.32	0.34	0.45	0.35	0.35	0.35	1.66
	**1**		0.35		0.34	0.45		0.34	0.35	
	**2**		0.35		0.34	0.4		0.36	0.34	
**1OSF**	**0**	0.09	0.38	0.04	0.05	0.29	0.68	0.72	0.31	1.58
	**1**		0.62		0.05	0.33		0.83	0.11	
	**2**		0.56		0.05	0.44		0.62	0.44	
**1PKF**	**0**	0.15	0.43	0.13	0.22	0.57	0.2	0.44	0.22	1.41
	**1**		0.79		0.2	0.57		0.41	0.41	
	**2**		0.7		0.19	0.46		0.39	0.21	
**1QY8**	**0**	0.2	0.07	0.06	0.07	0.41	0.25	0.07	0.07	1.51
	**1**		0.07		0.07	0.41		0.07	0.07	
	**2**		0.07		0.07	0.41		0.07	0.07	
**1S22**	**0**	0.25	1.06	0.17	0.52	0.41	0.41	1.1	0.25	1.71
	**1**		0.56		0.19	0.41		1.14	0.4	
	**2**		0.98		0.24	0.41		1.13	0.48	
**1S9D**	**0**	0.08	0.07	0.08	0.08	0.08	0.08	0.08	0.08	0.95
	**1**		0.08		0.08	0.08		0.08	0.08	
	**2**		0.08		0.08	0.08		0.08	0.08	
**1TPS**	**0**	0.27	0.55	0.11	0.49	0.25	0.47	0.72	0.1	1.4
	**1**		0.79		0.18	0.25		0.53	0.51	
	**2**		0.68		0.36	0.25		0.35	0.44	
**2ASP**	**0**	0.22	0.72	0.17	0.57	0.76	0.52	0.62	0.21	1.92
	**1**		0.14		0.2	0.88		0.37	0.64	
	**2**		0.44		0.53	0.79		0.19	0.22	
**2C6H**	**0**	0.12	0.04	0.04	0.04	0.1	0.4	0.04	0.04	0.74
	**1**		0.04		0.06	0.1		0.04	0.04	
	**2**		0.04		0.04	0.1		0.04	0.04	
**2DG4**	**0**	0.37	0.18	0.18	0.37	0.85	0.27	0.26	0.31	1.51
	**1**		0.27		0.19	0.93		0.27	0.29	
	**2**		0.19		0.53	0.93		0.29	0.32	
**2E9U**	**0**	0.52	0.4	0.34	0.4	0.39	0.53	0.4	0.4	0.95
	**1**		0.4		0.4	0.39		0.4	0.4	
	**2**		0.4		0.4	0.39		0.4	0.4	
**2ESA**	**0**	0.12	0.43	0.07	0.07	0.12	0.1	0.48	0.07	1.49
	**1**		0.92		0.07	0.12		0.31	0.11	
	**2**		0.43		0.1	0.12		0.46	0.31	
**2F3F**	**0**	0.15	0.34	0.13	0.16	0.16	0.17	0.15	0.16	0.72
	**1**		0.38		0.16	0.16		0.14	0.13	
	**2**		0.18		0.42	0.16		0.17	0.38	
**2HFK**	**0**	0.08	0.08	0.07	0.08	0.08	0.08	0.08	0.08	0.82
	**1**		0.08		0.08	0.08		0.08	0.08	
	**2**		0.08		0.08	0.08		0.08	0.08	
**2IWX**	**0**	0.15	0.14	0.14	0.14	0.26	0.51	0.14	0.14	1.53
	**1**		0.14		0.28	0.26		0.14	0.14	
	**2**		0.15		0.14	0.26		0.14	0.14	
**2IYA**	**0**	0.13	0.1	0.1	0.08	0.15	0.46	0.11	0.19	0.79
	**1**		0.1		0.11	0.15		0.53	0.11	
	**2**		0.1		0.15	0.15		0.48	0.19	
**2J9M**	**0**	0.06	0.06	0.06	0.06	0.07	0.35	0.06	0.06	0.59
	**1**		0.06		0.06	0.07		0.06	0.06	
	**2**		0.06		0.06	0.07		0.06	0.06	
**2PH8**	**0**	0.34	0.34	0.33	0.33	0.34	0.35	0.34	0.33	1.08
	**1**		0.34		0.34	0.34		0.33	0.34	
	**2**		0.33		0.34	0.34		0.34	0.34	
**2QZK**	**0**	0.21	0.21	0.15	0.21	0.19	0.21	0.21	0.21	1.33
	**1**		0.21		0.21	0.19		0.21	0.21	
	**2**		0.21		0.21	0.19		0.21	0.21	
**2WEA**	**0**	0.08	0.04	0.04	0.04	0.03	0.04	0.05	0.04	0.69
	**1**		0.04		0.04	0.03		0.04	0.04	
	**2**		0.04		0.04	0.03		0.04	0.04	
**2XBK**	**0**	0.32	0.36	0.26	0.36	0.45	0.38	0.37	0.35	1.23
	**1**		0.36		0.37	0.45		0.41	0.36	
	**2**		0.36		0.35	0.45		0.38	0.39	
**2XYT**	**0**	0.14	0.12	0.12	0.12	0.1	0.13	0.12	0.12	1.49
	**1**		0.12		0.12	0.1		0.12	0.12	
	**2**		0.12		0.12	0.1		0.12	0.12	
**3ABA**	**0**	0.24	0.22	0.21	0.24	0.71	0.24	0.21	0.22	1.31
	**1**		0.23		0.23	0.65		0.22	0.22	
	**2**		0.23		0.22	0.7		0.23	0.23	
**3BE9**	**0**	0.15	0.15	0.15	0.15	0.14	0.15	0.15	0.15	0.76
	**1**		0.15		0.15	0.14		0.15	0.15	
	**2**		0.15		0.15	0.14		0.15	0.15	
**3BXR 15**	**0**	0.08	0.05	0.05	0.06	0.65	0.07	0.06	0.05	0.65
	**1**		0.11		0.06	0.65		0.06	0.05	
	**2**		0.06		0.06	0.66		0.06	0.06	

**3BXR 16**	**0**	0.18	0.15	0.11	0.15	0.46	0.15	0.16	0.16	0.69
	**1**		0.39		0.14	0.45		0.16	0.14	
	**2**		0.37		0.15	0.45		0.15	0.16	
**3BXS A**	**0**	0.23	0.23	0.23	0.23	0.35	0.29	0.23	0.23	1.26
	**1**		0.23		0.23	0.35		0.23	0.23	
	**2**		0.23		0.23	0.35		0.23	0.23	
**3BXS B**	**0**	0.13	0.13	0.13	0.13	0.09	0.15	0.13	0.13	0.82
	**1**		0.13		0.13	0.09		0.13	0.13	
	**2**		0.13		0.13	0.09		0.13	0.13	
**3DV1**	**0**	0.12	0.54	0.11	0.14	0.49	0.13	0.15	0.15	0.89
	**1**		0.46		0.14	0.55		0.13	0.14	
	**2**		0.35		0.13	0.51		0.12	0.14	
**3DV5**	**0**	0.17	0.5	0.14		0.57	0.47	0.53	0.46	0.78
	**1**		0.49		0.46	0.56		0.49	0.47	
	**2**		0.56		0.47	0.56		0.48	0.46	
**3EKS**	**0**	0.03	0.01	0.01	0.01	0.05	0.01	0.01	0.01	0.38
	**1**		0.01		0.01	0.05		0.01	0.01	
	**2**		0.01		0.01	0.05		0.01	0.01	
**3FRQ**	**0**	0.16	0.07	0.07	0.07	0.1	0.13	0.07	0.08	0.84
	**1**		0.07		0.08	0.1		0.08	0.09	
	**2**		0.07		0.07	0.1		0.09	0.08	
**3I6O**	**0**	0.13	0.05	0.02	0.02	0.1	0.03	0.05	0.02	0.48
	**1**		0.07		0.04	0.07		0.03	0.03	
	**2**		0.07		0.03	0.07		0.03	0.03	
**3JRX**	**0**	0.14	0.13	0.11	0.12	0.16	0.13	0.12	0.12	1.24
	**1**		0.42		0.12	0.16		0.13	0.11	
	**2**		0.41		0.12	0.16		0.43	0.12	
**3K5C**	**0**	0.19	0.12	0.04	0.12	0.19	0.13	0.11	0.1	0.58
	**1**		0.11		0.12	0.19		0.12	0.12	
	**2**		0.1		0.12	0.13		0.12	0.11	
**3SU0**	**0**	0.13	0.08	0.06	0.08	0.38	0.07	0.08	0.08	0.87
	**1**		0.09		0.08	0.38		0.14	0.08	
	**2**		0.07		0.08	0.38		0.09	0.08	
**4HUS**	**0**	0.41	0.36	0.37	0.48	0.74	0.5	0.35	0.37	1.89
	**1**		0.47		0.41	0.74		0.35	0.39	
	**2**		0.4		0.37	0.7		0.37	0.48	
**4NNR**	**0**	0.28	0.76	0.16	0.47	0.27	0.56	0.48	0.43	1.71
	**1**		1.21		0.5	0.27		0.57	0.49	
	**2**		0.58		0.57	0.53		1.02	0.53	

Table S16. Mean values of the conformer with the lowest Root Mean Square Deviation ($RMSD_{RING}$) in Ångtröm to the X-ray_{ppw} ring conformation (full data set).

				Mean Ring Atom $RMSD_{RING}$					
**METHOD NAME**	**ENERGY MINIMIZED X-RAY**	**MCMM**	**MCMM-EXHAUSTIVE**	**MCMM-ENHANCED**	**PRIME-MCS**	**MD/LLMOD**	**MTLMOD**	**MTLMOD-ENHANCED**	**STARTING CONFORMER**
**PDB CODE**									
**1BXO**	0.28	0.19	0.18	0.19	0.30	0.19	0.20	0.19	0.64
**1EHL**	0.12	0.12	0.10	0.12	0.49	0.13	0.12	0.12	0.95
**1ESV**	0.10	0.10	0.10	0.10	0.42	0.16	0.10	0.10	1.22
**1FKD**	0.10	0.08	0.08	0.42	0.56	0.50	0.30	0.22	0.44
**1FKI**	0.16	0.61	0.17	0.64	0.63	0.63	0.67	0.57	2.26
**1LD8**	0.38	0.25	0.25	0.25	0.25	0.25	0.25	0.25	1.12
**1NMK**	0.09	0.30	0.08	0.33	0.47	0.19	0.76	0.41	1.66
**1NSG**	0.25	0.17	0.17	0.20	0.82	0.47	0.17	0.20	1.06
**1NT1**	0.36	0.35	0.32	0.34	0.43	0.35	0.35	0.35	1.66
**1OSF**	0.09	0.52	0.04	0.05	0.35	0.68	0.72	0.29	1.58
**1PKF**	0.15	0.64	0.13	0.20	0.53	0.20	0.41	0.28	1.41
**1QY8**	0.20	0.07	0.06	0.07	0.41	0.25	0.07	0.07	1.51
**1S22**	0.25	0.87	0.17	0.32	0.41	0.41	1.12	0.38	1.71
**1S9D**	0.08	0.08	0.08	0.08	0.08	0.08	0.08	0.08	0.95
**1TPS**	0.27	0.67	0.11	0.34	0.25	0.47	0.53	0.35	1.40
**2ASP**	0.22	0.43	0.17	0.43	0.81	0.52	0.39	0.36	1.92
**2C6H**	0.12	0.04	0.04	0.05	0.10	0.40	0.04	0.04	0.74
**2DG4**	0.37	0.21	0.18	0.36	0.90	0.27	0.27	0.31	1.51
**2E9U**	0.52	0.40	0.34	0.40	0.39	0.53	0.40	0.40	0.95
**2ESA**	0.12	0.59	0.07	0.08	0.12	0.10	0.42	0.16	1.49
**2F3F**	0.15	0.30	0.13	0.25	0.16	0.17	0.15	0.22	0.72
**2HFK**	0.08	0.08	0.07	0.08	0.08	0.08	0.08	0.08	0.82
**2IWX**	0.15	0.14	0.14	0.19	0.26	0.51	0.14	0.14	1.53
**2IYA**	0.13	0.10	0.10	0.11	0.15	0.46	0.37	0.16	0.79
**2J9M**	0.06	0.06	0.06	0.06	0.07	0.35	0.06	0.06	0.59
**2PH8**	0.34	0.34	0.33	0.34	0.34	0.35	0.34	0.34	1.08
**2QZK**	0.21	0.21	0.15	0.21	0.19	0.21	0.21	0.21	1.33
**2WEA**	0.08	0.04	0.04	0.04	0.03	0.04	0.04	0.04	0.69
**2XBK**	0.32	0.36	0.26	0.36	0.45	0.38	0.39	0.37	1.23
**2XYT**	0.14	0.12	0.12	0.12	0.10	0.13	0.12	0.12	1.49
**3ABA**	0.24	0.23	0.21	0.23	0.69	0.24	0.22	0.22	1.31
**3BE9**	0.15	0.15	0.15	0.15	0.14	0.15	0.15	0.15	0.76
**3BXR 15**	0.08	0.07	0.05	0.06	0.65	0.07	0.06	0.05	0.65
**3BXR 16**	0.18	0.30	0.11	0.15	0.45	0.15	0.16	0.15	0.69
**3BXS A**	0.23	0.23	0.23	0.23	0.35	0.29	0.23	0.23	1.26
**3BXS B**	0.13	0.13	0.13	0.13	0.09	0.15	0.13	0.13	0.82
**3DV1**	0.12	0.45	0.11	0.14	0.52	0.13	0.13	0.14	0.89
**3DV5**	0.17	0.52	0.14	0.46	0.56	0.47	0.50	0.46	0.78
**3EKS**	0.03	0.01	0.01	0.01	0.05	0.01	0.01	0.01	0.38
**3FRQ**	0.16	0.07	0.07	0.07	0.10	0.13	0.08	0.08	0.84
**3I6O**	0.13	0.06	0.02	0.03	0.08	0.03	0.04	0.03	0.48
**3JRX**	0.14	0.32	0.11	0.12	0.16	0.13	0.23	0.12	1.24
**3K5C**	0.19	0.11	0.04	0.12	0.17	0.13	0.12	0.11	0.58
**3SU0**	0.13	0.08	0.06	0.08	0.38	0.07	0.10	0.08	0.87
**4HUS**	0.41	0.41	0.37	0.42	0.73	0.50	0.36	0.41	1.89
**4NNR**	0.28	0.85	0.16	0.51	0.36	0.56	0.69	0.48	1.71

Table S17. Energy difference between: the energy minimized X-ray conformer to the global energy minimum and between the conformer closest to the X-ray conformation and the global energy minimum (full data set).

PDB	MCMM exhaustive RMSD (Å)	energy for lowest RMSD conformer ($kJ\ mol^{-1}$)	energy minimized X-ray conformation	lowest energy ($kJ\ mol^{-1}$)	energy difference (global energy minimum – lowest RMSD conformer)		energy difference (global energy minimum – energy minimized X-ray)	
					$kJ\ mol^{-1}$	$kcal\ mol^{-1}$	$kJ\ mol^{-1}$	$kcal\ mol^{-1}$
1BXO	0.59	-700.5	-733.2	-760.5[a]	**60.0**	14.3	27.3	6.5
1EHL	0.14	-568.1	-587.2	-601.6	33.5	8.0	14.4	3.4
1ESV	0.22	-490.2	-493.3	-505.6	15.4	3.7	12.4	3.0
1FKD	0.3	165.3	157.2	131.2	34.1	8.2	26.0	6.2
1FKI	0.42	235.1	223.5	207.6	27.5	6.6	16.0	3.8
1LD8	0.54	245.1	236.6	213.2[a]	31.9	7.6	23.4	5.6
1NMK	0.43	-385.3	-414.4	-451.3[a]	**66.0**	15.8	36.9	8.8
1NSG	0.3	54.6	60.5	38.3	16.2	3.9	22.1	5.3
1NT1	0.38	-274.6	-291.4	-294.7	20.0	4.8	3.2	0.8
1OSF	0.47	3.5	-9.7	-37.9	41.4	9.9	28.2	6.7
1PKF	0.32	-303.7	-297.3	-331.0	27.4	6.5	33.7	8.1
1QY8	0.07	-379.5	-389.8	-396.1	16.6	4.0	6.4	1.5
1S22	0.59	-393.1	-403.7	-441.4	**48.3**	11.5	37.7	9.0
1S9D	0.21	-183.0	-181.0	-187.0	4.1	1.0	6.0	1.4
1TPS	1.22	-867.59	-873.0	-924.3	**56.7**	13.6	**51.3**	12.3
2ASP	0.53	-554.7	-563.5	-588.8[a]	34.0	8.1	25.3	6.0
2C6H	0.67	24.4	-5.6	-18.3	**42.7**	10.2	12.7	3.0
2DG4	0.44	102.8	104.8	79.5	23.3	5.6	25.3	6.0
2E9U	0.37	-219.2	-223.1	-230.5	11.3	2.7	7.3	1.7
2ESA	0.2	-130.1	-136.8	-147.2	17.1	4.1	10.3	2.5
2F3F	0.74	-250.7	-259.5	-286.0	35.3	8.4	26.4	6.3
2HFK	0.13	-76.0	-77.8	-84.6	8.5	2.0	6.7	1.6
2IWX	0.18	-357.1	-353.0	-377.3	20.3	4.9	24.4	5.8
2IYA	0.43	-98.1	-106.3	-133.9	35.8	8.6	27.6	6.6
2J9M	0.78	-847.0	-847.0	-847.0	0.0	0.0	0.1	0.0
2PH8	0.57	-390.9	-389.2	-404.1	13.2	3.2	14.9	3.6
2QZK	0.78	-94.5	-94.4	-102.6	8.0	1.9	8.1	1.9
2WEA	0.8	-288.3	-293.3	-315.9	27.7	6.6	22.6	5.4
2XBK	0.59	-1 151.3	-1 131.9	-1 189.2	37.9	9.1	**57.3**	13.7
2XYT	0.22	249.7	241.0	240.3	9.4	2.2	0.7	0.2
3ABA	0.41	-745.0	-747.9	-765.4	20.4	4.9	17.5	4.2
3BE9	0.54	-576.3	-625.1	-630.2	**53.9**	12.9	5.1	1.2
3BXR	0.56	-549.8	-572.7	-597.9	**48.1**	11.5	25.1	6.0
3BXS A	0.38	-330.9	-330.9	-331.4	0.4	0.1	0.5	0.1
3BXS B	0.37	-327.5	-328.2	-331.4	3.9	0.9	3.2	0.8
3DV1	0.79	-284.1	-302.4	-328.0	**43.9**	10.5	25.6	6.1
3DV5	0.77	-51.3	-51.3	-96.4	**45.1**	10.8	**45.1**	10.8
3EKS	0.25	-149.1	-153.4	-158.8	9.7	2.3	5.4	1.3
3FRQ	0.43	-69.8	-75.2	-104.8	35.0	8.4	29.7	7.1
3I6O	0.66	-281.2	-304.9	-338.2	**57.1**	13.6	33.4	8.0
3JRX	0.26	-13.9	1.1	-24.3	10.4	2.5	25.4	6.1
3K5C	0.7	-46.8	-60.0	-100.4	**53.6**	12.8	40.4	9.7
3SU0	0.71	-659.8	-706.9	-749.2[a]	**89.4**	21.4	**42.3**	10.1
4HUS	0.61	-403.4	-400.0	-415.7	12.2	2.9	15.7	3.8
4NNR	0.49	134.2	150.2	103.0	31.2	7.5	**47.3**	11.3
min	0.07	NR[b]	NR[b]	NR[b]	0	0	0.1	0
max	1.22	NR[b]	NR[b]	NR[b]	89.4	21.4	57.3	13.7
median	0.44	NR[b]	NR[b]	NR[b]	27.7	6.6	23.4	5.6
average	0.5	NR[b]	NR[b]	NR[b]	29.7	7.1	21.7	5.2

[a]Lowest energy minimum was generated by another method else than MCMM exhaustive. [b]Not relevant. Energy differences greater than 10 $kcal\ mol^{-1}$ (41.8 $kJ\ mol^{-1}$) are bolded.

References

(1) Schrödinger. MacroModel Command Reference Manual; New York, NY, 2017.

(2) Schrödinger Release 2017-1: QikProp, Schrödinger, LCC, New York, NY, 2017.

(3) Instant JChem 15.9.14.0, ChemAxon. Http://www.chemaxon.com/.

(4) Chang, G.; Guida, W. C.; Still, W. C. An Internal Coordinate Monte Carlo Method for Searching Conformational Space. *J. Am. Chem. Soc.* **1989**, *111*, 4379–4386.
